# Gibberellins orchestrate panicle architecture mediated by DELLA–KNOX signalling in rice

**DOI:** 10.1111/pbi.13661

**Published:** 2021-08-24

**Authors:** Su Su, Jun Hong, Xiaofei Chen, Changquan Zhang, Mingjiao Chen, Zhijing Luo, Shuwei Chang, Shaoxing Bai, Wanqi Liang, Qiaoquan Liu, Dabing Zhang

**Affiliations:** ^1^ Joint International Research Laboratory of Metabolic & Developmental Sciences State Key Laboratory of Hybrid Rice School of Life Sciences and Biotechnology Shanghai Jiao Tong University Shanghai China; ^2^ Key Laboratory of Plant Functional Genomics of the Ministry of Education College of Agriculture Yangzhou University Yangzhou China; ^3^ School of Agriculture, Food and Wine University of Adelaide Urrbrae SA Australia

**Keywords:** biosynthesis, Gibberellin, KNOX class 1, panicle architecture, rice, signalling pathway, SLR1

## Abstract

Panicle architecture is a key determinant of grain yield in cereals, but the mechanisms governing panicle morphogenesis and organ development remain elusive. Here, we have identified a quantitative trait locus (*qPA1*) associated with panicle architecture using chromosome segment substitution lines from parents Nipponbare and 9311. The panicle length, branch number and grain number of Nipponbare were significantly higher than CSSL‐9. Through map‐based cloning and complementation tests, we confirmed that *qPA1* was identical to *SD1* (*Semi Dwarf1*), which encodes a gibberellin 20‐oxidase enzyme participating in gibberellic acid (GA) biosynthesis. Transcript analysis revealed that *SD1* was widely expressed during early panicle development. Analysis of *sd1/osga20ox2* and *gnp1/ osga20ox1* single and double mutants revealed that the two paralogous enzymes have non‐redundant functions during panicle development, likely due to differences in spatiotemporal expression; *GNP1* expression under control of the *SD1* promoter could rescue the *sd1* phenotype. The DELLA protein SLR1, a component of the GA signalling pathway, accumulated more highly in *sd1* plants. We have demonstrated that SLR1 physically interacts with the meristem identity class I KNOTTED1‐LIKE HOMEOBOX (KNOX) protein OSH1 to repress OSH1‐mediated activation of downstream genes related to panicle development, providing a mechanistic link between gibberellin and panicle architecture morphogenesis.

## Introduction

Rice (*Oryza sativa*) is one of the most important crops in the world, comprising the staple diet for over half of the world’s population. For decades, research has focused on improving rice yield to meet the demands of a rapidly growing population. A key determinant of grain yield in rice is spikelet numbers per panicle, which is directly regulated by inflorescence architecture; inflorescence meristems at the shoot apex differentiate into primary branch meristems attached to a central rachis, which then form several secondary branch meristems that bear spikelets (Zhang and Yuan, 2014). Several genes involved in inflorescence meristem development have been characterized, such as *aberrant spikelet and panicle1* (*ASP1*), *DROUGHT AND SALT TOLERANCE* (*DST*)*, TAWAWA1* (*TAW1*), and *ABERRANT PANICLE1* and *2* (*APO1 and APO2*), whose loss of function result in change of primary/secondary branch number, grain number or panicle length (Ikeda‐Kawakatsu *et al*., 2012; Ikeda‐Kawakatsu *et al*., 2009; Li *et al*., 2013; Yoshida *et al*., 2012; Yoshida *et al*., 2013). Researchers have also identified dozens of quantitative trait loci (QTL) that contribute to panicle morphology and grain yield. In japonica rice varieties, some examples include the following: *Dense and Erect Panicle1* (*DEP1*), which encodes the γ‐subunit of a heterotrimeric G‐protein complex that regulates meristem activity (Huang *et al*., 2009); and *OsSPL14*, also known as *Ideal Plant Architecture 1* (*IPA1*), a member of the SQUAMOSA PROMOTER BINDING PROTEIN‐LIKE (SPL) family of transcription factors that directly bind promoters of hormone synthesis and signalling‐related genes such as *SLENDER RICE 1* (*SLR1*) and *LONELY GUY* (*LOG*) (Lu *et al*., 2013). In indica varieties, *NARROW LEAF1* (*NAL1*) increases spikelet number and has become a useful breeding tool in indica‐growing regions such as South and South‐East Asia (Zhang *et al*., 2014). Recently, some of the genes in these QTLs have been identified through sequencing, bioinformatics and natural allelic diversity analysis (Zhang and Yuan, 2014), but further characterization of the genetic control of rice panicle shape is required to advance our fundamental understanding of biology to produce breeding innovation.

Gibberellic acid (GA) phytohormones are known to affect different plant developmental processes such as stem elongation, flowering, pollen maturation and seed germination (Cheng *et al*., 2004; King and Evans, 2003; Kuroha *et al*., 2018; Tyler *et al*., 2004). Four genes encoding GA 20‐oxidases (OsGA20ox1–4) have been thought to play a role in the penultimate step of GA biosynthesis (Spielmeyer *et al*., 2002). *SEMI‐DWARF 1* (*SD1*), known as the ‘Green Revolution’ gene, encodes GA 20‐oxidase 2, which has been applied in rice breeding for decades. Recently, *SD1* has been reported to modulate rice growth and grain yield by regulating nitrogen and carbon metabolism (Li *et al*., 2018). Moreover, *Grain Number per Panicle* (*GNP1*) has been isolated in rice that encodes a GA 20‐oxidase (Wu *et al*., 2016). Genetic variations in its promoter region affect transcript expression and panicle grain number, revealing a new role for GAs in rice inflorescence meristem development (Wu *et al*., 2016).

DELLA proteins are highly conserved plant‐specific GRAS family transcription regulators that usually act as negative regulators in the GA signalling pathway (Zentella *et al*., 2007). The DELLA N‐terminal domain binds with the GA receptor GIBBERELLIN INSENSITIVE 1 (GID1) to sense GA signalling (Daviere and Achard, 2016). DELLA proteins lack DNA‐binding elements, but interact with a diverse range of regulatory proteins – transcription factors, transcriptional regulators, chromatin remodelling complexes and co‐chaperones – to regulate downstream processes (Van De Velde *et al*., 2017). A single DELLA protein, SLR1/OsGAI, has been reported in rice (Ikeda *et al*., 2001; Ogawa *et al*., 2000), which interacts directly with NAC transcription factors to inhibit a NAC–MYB–CESA signalling cascade that regulates secondary cell wall cellulose synthesis (Huang *et al*., 2015). SLR1 can also disrupt nitrogen metabolism, by disrupting the interaction between the transcription activator GRF4 and its co‐factor GIF, which promotes nitrogen uptake and assimilation (Li *et al*., 2018). Class 1 KNOX subfamily proteins are three‐amino acid loop extension (TALE) homeodomain (HD) transcription factors involved in establishment and maintenance of the shoot apical meristem (SAM) (Scofield *et al*., 2007; Sentoku *et al*., 1999; Vollbrecht *et al*., 1991). Five functional class 1 *KNOX* genes (*OSH1*, *OSH6*, *OSH15*, *OSH43* and *OSH71*) have been identified in rice (Sato *et al*., 1999; Sentoku *et al*., 1999). KNOX class 1 proteins can homo‐ or heterodimerize with other HD proteins to affect development and growth (Bellaoui *et al*., 2001; Bhatt *et al*., 2004; Muller *et al*., 2001), and directly regulate phytohormone biosynthesis and metabolism to create the correct hormone balance to form and maintain the SAM (Sakamoto *et al*., 2006). *OSH1* is required for SAM maintenance after germination, whereas double *osh1 osh15* mutants lack a SAM during embryogenesis and regeneration (Tsuda *et al*., 2011).

In this study, we have identified a QTL, *qPA1* (QTL for panicle architecture on chromosome 1), which regulates rice panicle architecture. Through map‐based cloning and complementation tests, we show that *qPA1* is identical to *SD1*, which positively regulates rice panicle length and spikelet number via the DELLA–class 1 KNOX pathway. This work provides insight into rice panicle development and provides new breeding targets for panicle architecture refinement. In addition, combining these two QTLs (*GNP1* and *SD1*) provides new breeding targets for panicle architecture refinement.

## Results

### Map‐based cloning of *qPA1* reveals the underlying *SD1* gene

**Figure 1:**
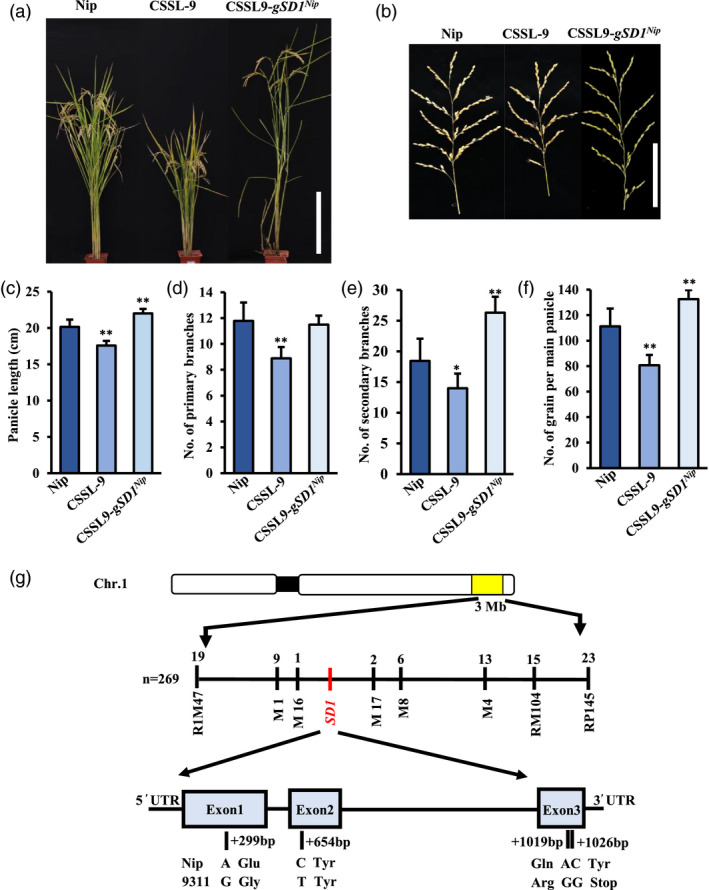
Phenotype of parental, CSSL‐9 and complemented plants, and map‐based cloning of *qPA1*. (a) Plant architecture of Nipponbare (Nip), CSSL‐9 and complemented plants. Bar = 30 cm. (b) Panicle architecture of Nip, CSSL‐9 and complemented plants. Bar = 10 cm. (c–f) Panicle traits of Nip, CSSL‐9 and complemented plants, showing (c) panicle length; number of (d) primary and (e) secondary branches per panicle; and (f) number of grains in the main panicle. Mean ± SE, *n* = 20. (g) Map‐based cloning of the plant height QTL on chromosome 1 in the CSSL‐9 × Nipponbare F_2_ population, showing the position and result of the 4 SNPs. Differences in wild‐type plants indicated *P < 0.05 and **P < 0.01, *t*‐test.

Among 136 chromosome segment substitution lines (CSSLs) derived from two inbred parents – Nipponbare (receptor parent) and 9311 (donor parent) – only CSSL‐9 exhibited short stature and small panicle size with reduced primary and secondary branch numbers compared with the receptor parent (Figure 1a–f; Figure S1; Zhang *et al*., 2011). The number of primary and secondary branches per panicle of CSSL‐9 decreased by ~17% and ~20%, respectively, compared with the Nipponbare parent (Figure 1d, e), which resulted in a ~23% reduction in grain number in the main panicle (Figure 1f). CSSL‐9 was found to harbour an introgression segment from 9311 on chromosome 1 between two markers R1M147 and RP145 (Figure 1g).

To determine the gene(s) underlying the CSSL‐9 phenotype, an F_2_ segregation population derived from CSSL‐9 and Nipponbare was constructed for fine mapping. Short stature and small panicle size were observed to co‐segregate, suggesting that these two traits are controlled by a single QTL, designated *qPA1*. Panicle size of the F_2_ population segregated as small:large 263:838 (χ^2^ = 0.669 < $\chi_{2,0.05}^{2}$ = 3.841_,_ *P* > 0.05), indicating that *qPA1* is a single locus that segregates in a Mendelian ratio. Using markers, the candidate region was narrowed down to the region flanked by M16 and M17, which contains 43 genes based on the Rice Genome Annotation Project Database (http://rice.plantbiology.msu.edu/) (Figure 1g). Of these, LOC_Os01g66100, also known as *SD1*, encodes a putative GA 20‐oxidase 2 with a known semi‐dwarf and yield‐related effect, and was thus a strong candidate gene for *qPA1*.

To examine whether *SD1* is responsible for the *qPA1* phenotype, the Nipponbare genomic *SD1* sequence was introduced into CSSL‐9. All 20 T_1_ transformants (complemented lines) showed significantly longer panicle length, higher plant height, increased primary and secondary branch numbers, and total grain number per panicle than CSSL‐9 lines (Figure 1a–f), confirming that *SD1* is the causal gene for *qPA1*.

### Small panicle size is caused by a non‐functional *SD1^9311^* allele

To find the genetic cause of smaller panicle size in CSSL‐9, we sequenced *SD1* (LOC_Os01g66100) in both parents. Genomic analysis of *SD1* in CSSL‐9/9311 and Nipponbare lines revealed 74 single nucleotide polymorphisms (SNPs) in the 2 kb promoter region, 41 SNPs in introns and four SNPs in exons: 2 mis‐sense mutations at +299 and +1019 bp, one silent mutation at +654 bp and a non‐sense mutation at +1026 bp that truncates the protein by 47 amino acids (Figure 1g).

**Figure 2:**
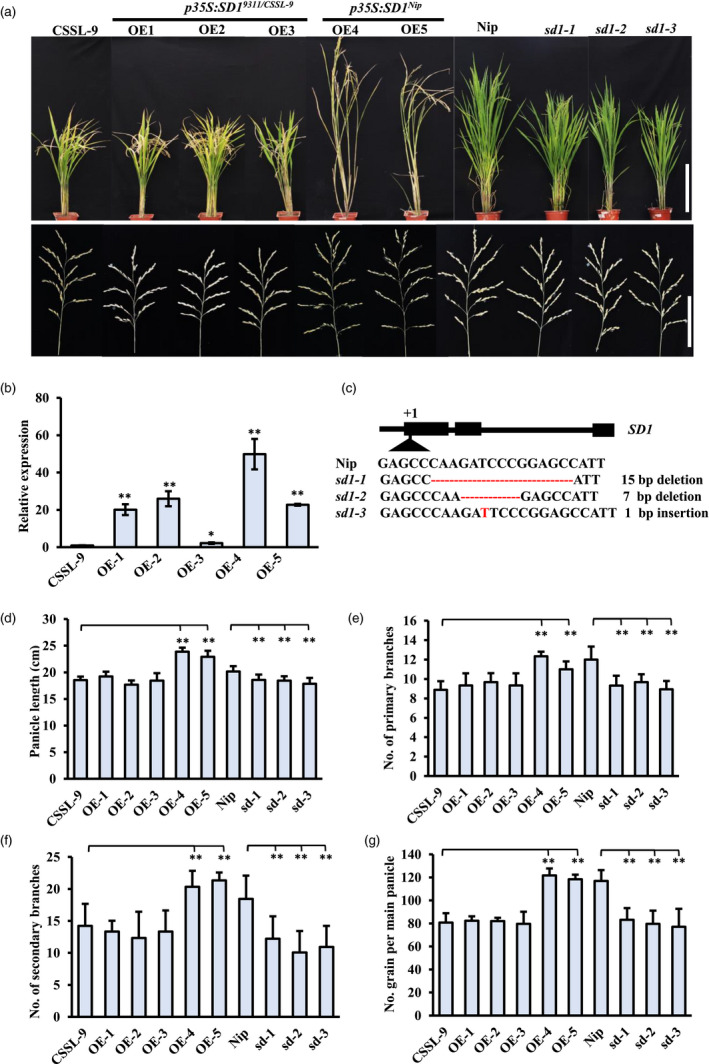
Phenotype of *SD1* transformants. (a) Plant architecture of overexpression lines, *sd1* mutants, and control plants. Nip, Nipponbare; OE, overexpression lines in CSSL‐9; *sd1*, *SD1* knockout lines. For plant architecture, Bar = 30cm; For panicle architecture, Bar = 10cm.(b) Relative expression of *SD1* in transgenic inflorescence primordia. Mean ± SE, *n* = 3. (c) Target sequences of CRISPR/Cas9‐mediated *sd1* knockout lines. (d–g) Panicle traits of overexpression lines, *sd1* mutants, and control plants, showing (d) panicle length; number of (e) primary and (f) secondary branches per panicle; and (g) number of grains in the main panicle. Mean ± SE, *n* = 20. Differences in CSSL‐9 plants indicated *P < 0.05, **P < 0.01, *t*‐test.

To determine the functions of Nipponbare and 9311 alleles, we transformed CSSL‐9 lines with overexpression constructs encoding different parental alleles under control of the CaMV 35S promoter (Figure 2a). *SD1* expression levels in all transgenic plants were higher than in the CSSL‐9 control line (Figure 2b). Transgenic lines expressing the Nipponbare allele (*SD1^Nip^*) were taller and had significantly increased panicle length, primary and secondary branch numbers, and total grain number than the CSSL‐9 line (Figure 2a, b, d–g). In contrast, no obvious change in panicle traits was observed in transgenic lines overexpressing the 9311 allele (*SD1^9311^*), suggesting that the *SD1^9311^* allele may be non‐functional (Figure 2d–g).

According to the previous research, *SD1^Nip^* is an *SD1‐EQ‐*type allele with a weaker phenotype than a *SD1‐GR* allele present in other rice varieties such as Kasalath (Asano *et al*., 2011). By comparing coding sequences of *SD1* in Kasalath (*SD1^Kas^*) with *SD1^9311^*, we found two SNPs: the same silent mutation at +654 bp as for *SD1^Nip^*, and the non‐sense mutation at +1026 bp (Figure S2). The two nucleotide changes at +299 and +1019 bp, which caused mis‐sense mutations between *SD1^9311^* and *SD1^Nip^*, were identical in *SD1^Kas^* and *SD1^9311^*. These codons define the stronger *SD1‐GR* allele *SD1^Kas^*, indicating that these mutations do not compromise protein function. Thus, the only difference between *SD1^9311^* and *SD1^Nip^* that could lead to a non‐functional *SD1^9311^* allele was the non‐sense mutation at +1026 bp that led to premature truncation of the protein. This genetic variation is rare in natural accessions, with only 21 varieties in the 3K genome data having this variation (Table S2) (Mansueto *et al*., 2017), which does not appear advantageous for rice growth and development under selection pressure.

To examine the effects of SNPs in the promoter region, we examined *SD1* expression in CSSL‐9 and Nipponbare. Gene expression was higher in CSSL‐9 than in Nipponbare plants (Figure S3a), supported by higher observed transcriptional activity of the *SD1^9311^* promoter as measured by a dual‐luciferase assay in *Nicotiana benthamiana* (Figure S3b). Thus, differences in gene expression due to divergent promoter sequences cannot explain the lack of function of *SD1* in CSSL‐9, further supporting our conclusion that small panicle size in CSSL‐9 is caused by the single non‐sense mutation in the *SD1* coding region.

### *SD1* play a crucial role in panicle development

To determine the effects of *SD1* in different backgrounds, CRISPR/Cas9 was applied to knock out *SD1* in Nipponbare and Kasalath lines (Figure 2a, c; Figure S4). Plant height was decreased in both backgrounds, shown in Kasalath due to decreases in length of all internodes (Figure 2a; Figure S4c). The panicle length of *sd1* homozygous mutants was reduced by 8–21%, the primary branch number by 18–26%, the secondary branch number by 34–46% and the total grain number per main panicle by 29–41% (Figure 2d–g; Figure S4d–g). These results indicate the loss of *SD1* function resulted in smaller, less branched panicles, resulting in decreased yield in two backgrounds. Even *SD1* in Nipponbare is a weak allele, it still has an indispensable role in panicle shape and plant height. Combing these with overexpression *SD1^Nip^* transgenic plants produced a larger panicle (Figure 2), *SD1* likely plays a positive role in panicle architecture.

**Figure 3:**
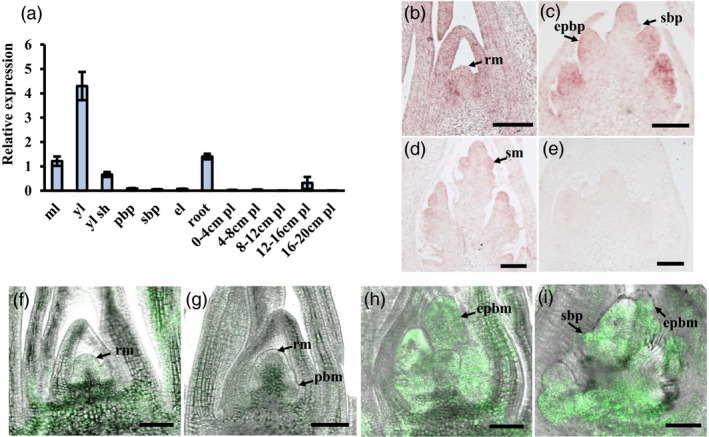
*SD1* expression in wild‐type tissues and during SAM development. (a) Relative *SD1* expression in tissues during rice development. Mean ± SE, *n* = 3. ml, mature leaf; yl, young leaf; yl sh, young leaf sheath; pbp, primary branch primordium; sbp, secondary branch primordium; el, elongating stem; pl, panicle length. (b–e) RNA *in situ* assay for *SD1* expression (b) at rachis meristem stage; (c) primary branch primordium later stage and secondary branch primordium initial stage; (d) secondary branch primordium later stage; and (e) sense probe as negative control at primary branch stage. Bar = 100 μm. (f–i) GFP signal directed by the *SD1* promoter at different stages of branch primordium development, including (f) rachis meristem stage; (g) primary branch primordium initial stage; (h) primary branch primordium later stage; (i) secondary branch primordium initial stage. Bar = 100 μm. rm, rachis meristem; pbm, primary branch meristem; ebpb, elongating primary branch meristem; sm, spikelet meristem.

*SD1* has previously been shown to be expressed globally throughout plant development, especially in vegetative organs (Monna *et al*., 2002; Sakamoto *et al*., 2004), in line with our qRT‐PCR results showing expression in leaf, leaf sheath, roots and panicle (Figure 3a). *In situ* hybridization was performed to determine the spatial expression profile of *SD1* more precisely during panicle development. *SD1* was detected in rachis meristems, elongated primary and secondary branch meristems, and spikelet meristems (Figure 3b–e). Similarly, GFP localization under control of the 3‐kb *SD1* promoter in transgenic plants was observed in these tissues of rachis meristem, early primary branch meristem, elongated primary branch meristem and secondary branch meristem (Figure 3f–i). These results indicate that *SD1* expresses during early panicle development.

### Divergent functions of *SD1* and *GNP1* are due to changes in promoter activity

Phylogenetic analysis of GA 20‐oxidases from five monocotyledons and one dicotyledon revealed that OsGA20ox1/GNP1 and OsGA20ox3 clustered in a large clade that contained most Arabidopsis proteins, while OsGA20ox2/SD1 and OsGA20ox4 belonged to a separate clade (Figure S5). Each OsGA20ox paralogue falls in the same clade with GA20oxs from other monocotyledon species.

**Figure 4:**
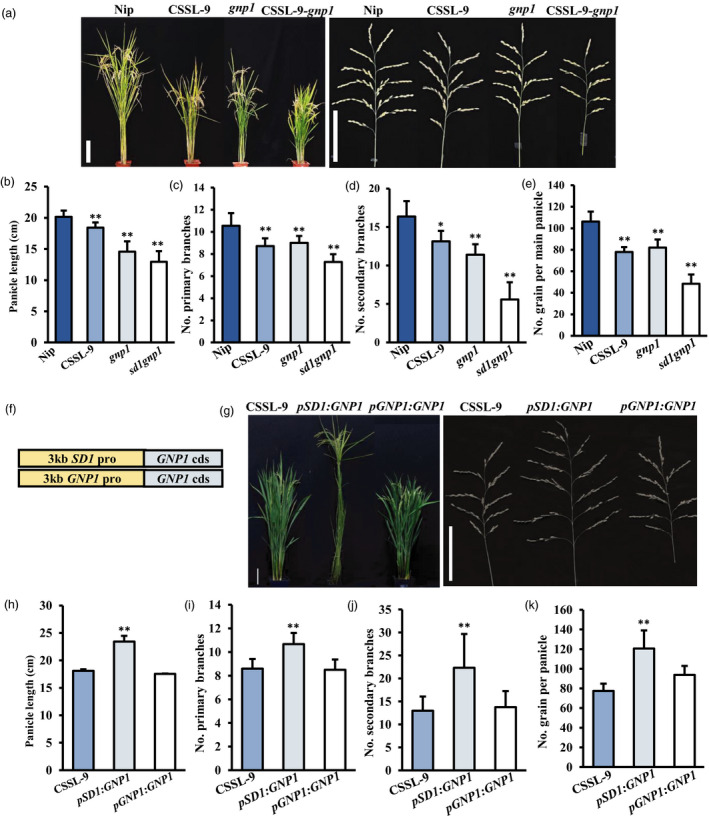
Phenotypes of knockout mutants and complemented plants of *GNP1*. (a) Plant and panicle architecture of *sd1* and *gnp1* single and double mutants. Bar = 10 cm. (b–e) Panicle traits of single and double mutants, showing (b) panicle length; number of (c) primary and(d) secondary branches per panicle; and (e) number of grains in the main panicle. Mean ± SE, *n* = 20. (f) Gene structure with different promoters. (g) Plant architecture and panicle morphology of complemented plants with different promoters. Bar = 10 cm. (h–k) Panicle traits of complemented plants, showing (h) panicle length; number of (i) primary and (j) secondary branches per panicle; and (k) number of grains in the main panicle. Mean ± SE, *n* = 3. Differences in wild‐type plants indicated *P < 0.05 and **P < 0.01, *t*‐test.

In rice, *OsGA20ox1/GNP1* and *OsGA20ox2/SD1* have been found to control rice plant height and yield (Li *et al*., 2018; Wu *et al*., 2016). Both proteins were observed to localize to the cytoplasm (Figure S6). To study possible redundant functions of *GA20ox1* and *GA20ox2*, a *gnp1* mutation was generated in Nipponbare and CSSL‐9 backgrounds via CRISPR/Cas9 technology to create a *gnp1* single mutant and a *gnp1 sd1* double mutant, respectively (Figure 4). Compared with Nipponbare, *gnp1* single mutants showed a decrease of ~15% and ~30%, respectively, in primary and secondary branch numbers and ~23% reduction in grain number per panicle (Figure 4c–e). The double mutant *sd1gnp1* showed a more severe reduction: ~30%, ~65% and ~54% reduction in primary branch, secondary branches and total grain numbers per main panicle compared with Nipponbare (Figure 4c–e). These data suggest that *GNP1* and *SD1* may function independently to regulate panicle development, and cause a dosage effect of GA to influence plant development.

In Arabidopsis, AtGA20oxs exhibit partial redundancy, mainly due to differences in expression patterns (Plackett *et al*., 2012; Rieu *et al*., 2008). According to our qRT‐PCR results and the RiceXPro database (Sato *et al*., 2013), both *GNP1* and *SD1* are expressed throughout plant development, including during panicle development (Figure S7; Wu *et al*., 2016), but the *GNP1* was expressed at much higher levels during panicle development, while *SD1* was expressed more highly in vegetative organs such as young leaf and root (Figure S7). To investigate potential differences in transcriptional regulation of these two genes in rice, we transformed the coding sequence of *GNP1* under control of the *SD1* and *GNP1* promoters (*pSD1:GNP1* and *pGNP1:GNP1*, respectively) into CSSL‐9 (Figure 4f–k; Figure S8). While the *pGNP1:GNP1* construct had little effect on the CSSL‐9 phenotype, the *pSD1:GNP1* construct could rescue the *sd1* phenotype (Figure 4g–k). This result indicates that their spatiotemporal patterns of expression are associated with their functional divergence.

### GAs may regulate other genes known to be involved in panicle development

**Figure 5:**
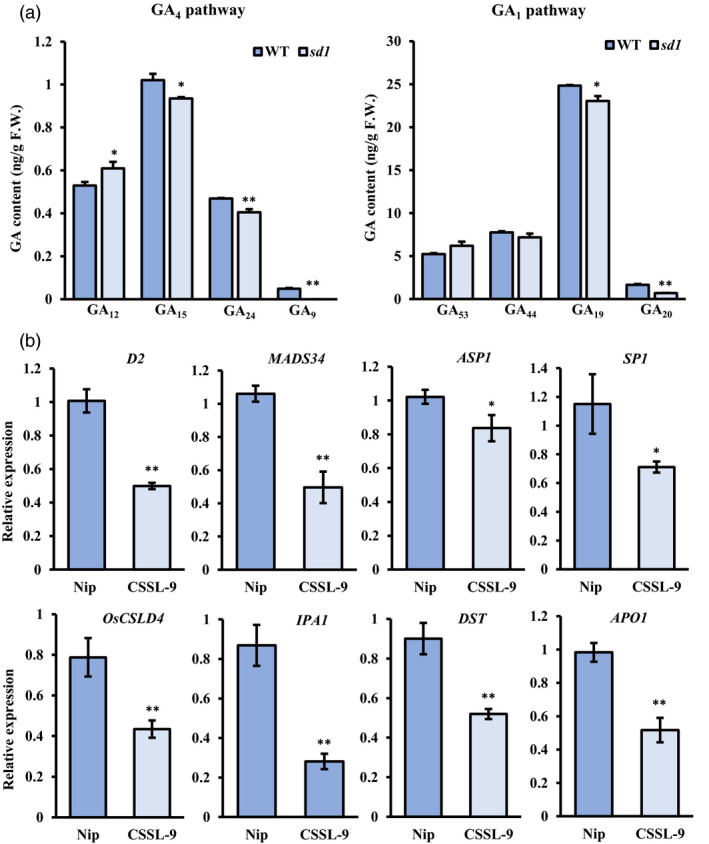
*SD1* allele affects GA biosynthesis and expression of panicle‐related genes. (a) GA content in *sd1* and wild‐type lines (Nipponbare) of eight GA biosynthesis intermediates during early panicle development (~1 cm). Mean ± SE, *n* = 3. F.W., fresh weight. (b) Relative expression of genes involved in panicle development in panicle branch primordia of wild‐type and *sd1* mutant plants. Mean ± SE, *n* = 3. Difference in wild‐type plants indicated: *P < 0.05 and **P < 0.01, *t*‐test.

GA 20‐oxidases participate in the GA biosynthetic process by converting GA_53_ to GA_20_ via GA_44_ and GA_19_, or by converting GA_12_ to GA_9_ via GA_15_ and GA_24_ (Olszewski *et al*., 2002). Quantification of GAs in young panicles showed that levels of GA intermediates produced by OsGA20oxs were lower in *sd1* plants: levels of GA_15_, GA_24_ and GA_9_ in the GA_4_ pathway, and GA_19_ and GA_20_ in the GA_1_ pathway decreased significantly (Figure 5a), indicating that loss of *SD1* function impacted GA synthesis. However, bioactive GA_4_ was not detected in either wild‐type and *sd1* plants, while levels of bioactive GA_1_ and GA_3_ did not change (Figure S9), whereas the expression of GA catabolic enzymes *OsGA2ox2*, *OsGA2ox5* and *OsGA2ox6* was decreased significantly in *sd1* mutants (Figure S10). Therefore, we speculated that the similar level of GA_1_ and GA_3_ between wide‐type and *sd1* mutants was caused by down‐regulation of these catabolic genes in the *sd1* mutant.

The expression of known regulators of panicle development was examined in young panicle of *sd1* and wild‐type Nipponbare to assess whether their transcription altered in response to different levels of GA intermediates. Some genes, such as *D2* (*ebisu dwarf*) (Fang *et al*., 2016), *MADS34* (Gao *et al*., 2010), *ASP1* (Yoshida *et al*., 2012), *SP1* (Li *et al*., 2009), *OsCSLD4* (Luan *et al*., 2011), *IPA1* (Lu *et al*., 2013), *DST* (Li *et al*., 2013) and *APO1* (Ikeda‐Kawakatsu *et al*., 2009), showed decreased expression in *sd1* plants compared with wild type (Figure 5b). However, expression of other genes, such as *APO2* (Ikeda‐Kawakatsu *et al*., 2012), *DTH7* (Yan *et al*., 2013), *DEP1* (Sun *et al*., 2014), *TAW1* (Yoshida *et al*., 2013), *FUWA* (Chen *et al*., 2015), *FBK12* (Chen *et al*., 2013) and *GNP1* (Wu *et al*., 2016), was similar in both wild‐type and *sd1* plants (Figure S11). These results indicate that changes in GA synthesis, particularly levels of intermediate GA forms, may induce transcription changes in some genes related to panicle development.

### SLR1 and KNOX class 1 proteins interact directly via specific domains

**Figure 6:**
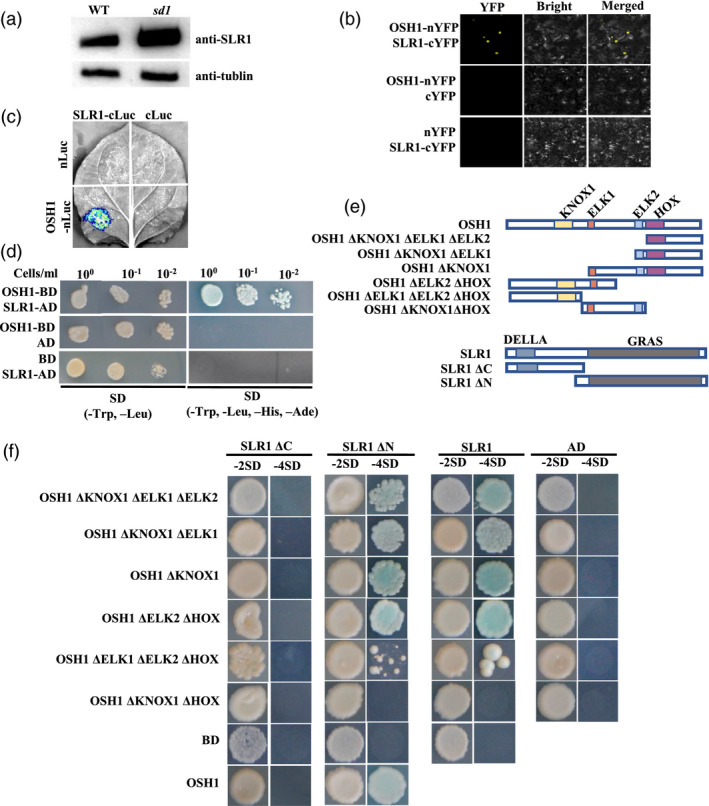
OSH1 can directly interact with SLR1. (a) SLR1 protein levels detected by Western blot in young panicle of wild‐type and s*d1* mutants. (b) OSH1 interacts with SLR1 in a BiFC assay. OSH1 was fused to the N‐terminal region of YFP (nYFP); SLR1 was fused to the C‐terminal region of YFP (cYFP). (c) Split‐luciferase assays between OSH1 and SLR1 with controls in tobacco leaves. cLuc, C‐terminal luciferase; nLuc, N‐terminal luciferase. (d) OSH1 interacts with SLR1 in Y2H assays. OSH1 was fused to the GAL4 binding domain (BD); SLR1 was fused to the GAL4 activation domain (AD). (e) Schematic diagrams of truncated OSH1 and SLR1 proteins. (f) Interactions between OSH1 and SLR1 truncated proteins. OSH1‐truncated proteins were fused to the GAL4 binding domain (BD); SLR1 was fused to the GAL4 activation domain (AD).

DELLA proteins have been reported to interact with a large number of proteins, such as OsYABBY4, WRKY45, NAC29/31 and class I TCP to mediate GA‐induced plant growth and development (Chen *et al*., 2017; Daviere and Achard, 2016; Daviere *et al*., 2014; Huang *et al*., 2015; Yang *et al*., 2016). The amount of the rice DELLA protein SLR1 increased dramatically in *sd1* compared with wild‐type plants (Figure 6a), further reinforcing the key role of GA signalling in panicle development. A yeast two‐hybrid (Y2H) assay was performed to identify proteins that may interact with SLR1 to regulate panicle development. We observed that KNOX class 1 proteins (OSH1, OSH6, OSH15, OSH43 and OSH71) could directly interact with SLR1 in Y2H system, and confirmed this result using split‐luciferase and bimolecular fluorescence complementation (BiFC) assays (Figure 6b–d; Figure S12).

*KNOX* class 1 genes have been implicated in inflorescence formation. Genes encoding these five KNOX class 1 proteins are expressed in shoot apical meristem, inflorescence meristem and floral meristem (Harrop *et al*., 2016; Sato *et al*., 1999; Sentoku *et al*., 1999), and their expression is induced by GAs (Wu *et al*., 2016). *OSH1*, *OSH6*, *OSH43*, *OSH15* and *OSH71* expressions were lower in *sd1* compared with wild‐type plants (Figure S13). As *osh1* mutants have a smaller panicle than wild‐type plants (Tsuda *et al*., 2011), we selected OSH1 for further protein domain analysis. OSH1 proteins are predicted to have four domains – a KNOX 1 domain, two ELK domains and a HOX domain – while DELLA proteins have two predicted domains, an N‐terminal DELLA domain and a C‐terminal GRAS domain (Figure 6e; http://smart.embl‐heidelberg.de/). In OSH1, the KNOX 1 and ELK domains are required for suppression of target gene expression, while the HOX domain is important for homodimerization and binding to target sequence (Nagasaki *et al*., 2001). Domains in both proteins were systematically deleted, singly and in combinations, to identify interacting domains (Figure 6e). The truncation interaction experiments in yeast showed that either the HOX or the KNOX 1 domain of OSH1 was required to mediate interaction with the SLR1 GRAS domain (Figure 6f).

**Figure 7:**
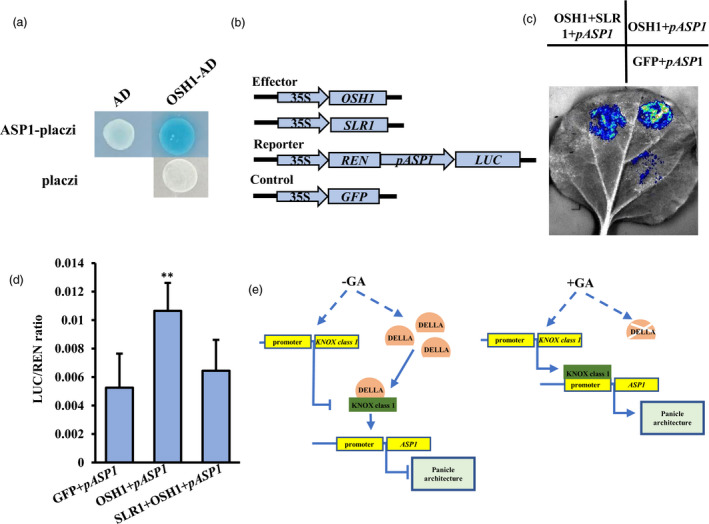
KNOX class 1 proteins work together with DELLA proteins to regulate expression of downstream genes. (a) OSH1 binds *ASP1* promoter in a Y1H assay. AD, activation domain; plazci, β‐glucuronidase reporter gene. (b–d) OSH1 binds to *ASP1* promoter in a luciferase reporter assay. Four constructs (b) were co‐expressed in different combinations in tobacco leaves (c), and normalized luciferase activity (d) quantified. Mean ± SE, *n* = 3, **P < 0.01, *t*‐test. (e) Model for GA regulation of panicle development in rice.

To examine whether SLR1 can repress gene activation by OSH1, we used reporter genes driven by the *OsREL2/ASP1* promoter; *ASP1* has been reported to regulate panicle architecture and contains putative OSH1 binding motifs in its promoter (Eric *et al*., 1995; Bolduc and Hake, 2009; Kwon *et al*., 2012; Nagasaki *et al*., 2001; Sakamoto *et al*., 2004; Tsuda *et al*., 2011; Tsuda *et al*., 2014; Yoshida *et al*., 2012). Yeast 1‐hybrid (Y1H) and luciferase assays confirmed that OSH1 could directly bind the *ASP1* promoter (Figure 7a) and that co‐expression of SLR1 with OSH1 reduced *ASP1* expression (Figure 7b–d).

Our results indicate that a DELLA protein (SLR1) can disrupt KNOX class 1 protein (OSH1) activation of downstream genes via physical interaction. When loss of function of *GA 20‐oxidase 2/SD1* accumulated, SLR1 interacts with KNOX class 1 proteins to represses KNOX class 1‐mediated activation of downstream genes. When *SD1* has normal function, DELLA proteins are decreased and cannot interact with KNOX class 1 proteins, promoting downstream gene expression to direct GA‐regulated panicle development (Figure 7e).

## Discussion

### *SD1* acts as a positive regulator determining panicle architecture

Here, we have used map‐based cloning of CSSLs and complementation experiments to identify the *qPA1* QTL as the *SD1* gene known to affect plant height and panicle development (Figure 1). The CSSL‐9 phenotype could be rescued by overexpression of the SD1^Nip^, but not the SD1^9311^, protein suggesting that SD1^9311^ in CSSL‐9 was not functional. Sequence comparison of the two parental alleles (japonica Nipponbare and indica 9311) revealed several SNPs in the promoter, introns and coding region (Figure 1g). The coding region contained two mis‐sense SNPs (at +299 bp and +1019 bp) that are known to produce two functional alleles of different ‘strengths’ (Asano *et al*., 2011), so the mutation that caused *SD1^9311^* loss of function was the premature termination that truncated the protein by 47 amino acids. Combing these with overexpression and knockout transgenic results, it indicates that *SD1* acts as a positive factor to determine panicle architecture.

SD1 catalyses conversion of GA_53_ and GA_12_ into GA_20_ and GA_9_, respectively, by multistep reactions; these GA intermediates are finally converted into functional GA_1_ and GA_4_, respectively, by a GA 3‐oxidase (Itoh *et al*., 2001; Kuroha *et al*., 2018). GA_1_ is predominant in vegetative tissues, while GA_4_ levels peak in anthers during reproductive development (Kuroha *et al*., 2018; Kobayashi *et al*., 1988; Zhu *et al*., 2006). However, the details of how GAs finely regulate rice panicle development remain unknown. In this study, we did not detect any changes in levels of bioactive GAs (GA_1_, GA_3_ and GA_4_) in *sd1* and wild‐type inflorescences (Figure S9); however, GA intermediates were significantly reduced in *sd1* compared with wild‐type plants (Figure 5a). Similarly, Rieu *et al*., (2008) found similar levels of GA_1_ and GA_4_ in wild‐type and *ga20ox1* Arabidopsis plants, although the double *ga20ox1 ga20ox2* mutant had significantly lower levels of GA_1_ and GA_4_. They reasoned that even minor changes in hormone levels could cause distinct changes in plant growth or that specific spatiotemporal changes in GA levels were not detected. However, the study of Wu *et al*., (2016) showed that the expression of *OsGA2ox*s could affect final GA contents. In our study, *OsGA2ox2*, *OsGA2ox5* and *OsGA2ox6* were decreased obviously in *sd1* mutants (Figure S10). It may indicate that down‐regulation of these catabolic genes results in similar GA_1_ and GA_3_ level between wide‐type and sd1 mutants.

As *SD1* is one of the OsGA20ox enzymes, its eventual products are GA_9_ and GA_20_. We suggest another possibility that precursors of known bioactive GAs may have some biological function during rice panicle development. We found that levels of GA_20_ and GA_9_, the ultimate products of SD1, were most affected in *sd1* lines, decreasing by ~58% and ~100%, respectively (Figure 5a). GA_9_ has been shown to be more effective than GA_3_ or GA_1_ in promoting interaction between SLR1 and the GA receptor (Ueguchi‐Tanaka *et al*., 2007); and more active than GA_1_ and GA_4_ in promoting stem growth in *Thiaspi arvense* L. (Metzger, 1990). However, more research will be required to provide evidence for our theory that GA intermediates play a critical role during rice panicle development.

### GNP1 and SD1 are functionally divergent due to different patterns of gene expression

GA 20‐oxidases have been reported to mediate GA‐regulated developmental processes in plants, affecting plant height, flowering and fertility (Ashikari *et al*., 2002; King and Evans, 2003; Rieu *et al*., 2008; Spielmeyer *et al*., 2002; Tyler *et al*., 2004). Phylogenetic analysis of GA 20‐oxidases from five cereal crops revealed that the GA20ox paralogues group together (Figure S5), consistent with the result of Plackett *et al*. (2012) who suggested that *GA20ox* gene duplication happened before the divergence. Arabidopsis GA20ox proteins were more similar to each other than to their respective cereal paralogues, suggesting considerable divergence between dicot and monocot proteins.

In rice, previous studies of *OsGA20ox1* and *OsGA20ox2* have identified effects on plant height and yield (Li *et al*., 2018; Oikawa *et al*., 2004; Sasaki *et al*., 2002; Wu *et al*., 2016). We have shown that *sd1* (*osga20ox2*) and *gnp1* (*osga20ox1*) single mutants both have significant decreased plant height and panicle traits, while the double mutants exhibited even more severe phenotypes (Figure 4a–e). Thus, these two genes also show similar but complementary roles in rice development. Functional differences between paralogues are most likely caused by three differences based on previous studies. Firstly, different subcellular localizations may lead to different functions, such as between the tonoplast‐located OsNRT1.1A and plasma membrane‐located OsNRT1.1B in nitrogen utilization (Wang *et al*., 2018). However, both SD1/OsGA20ox2 and GNP1/OsGA20ox1 were observed to localize in the cytoplasm (Figure S6). Secondly, different spatiotemporal patterns of the two genes may lead to their functional variation, such as *KNOX* Class 1 genes and *KNOX* class 2 genes (Kerstetter *et al*., 1994). *OsGA20ox1/GNP1* and *OsGA20ox2/SD1* had partially overlapping expression patterns throughout development: *SD1* was expressed more highly in young leaves and roots, while *GNP1* was expressed more highly in panicle organs (Figure S7). In *sd1* mutant, *GNP1* expression was not significantly changed (Figure S11). In addition, the results of Wu *et al*., (2016) showed that *SD1* expression was not changed between NIL‐*GNP1^LT^* and NIL‐*GNP1^TQ^*. These showed that *SD1* and *GNP1* may not show the antagonistic expression. It is likely that *SD1* and *GNP1* have differential roles for panicle, which remains to be investigated. While *GNP1* expressed under its own promoter could not rescue the *sd1* phenotype in CSSL‐9 plants, *GNP1* expression under an *SD1* promoter could (Figure 4f–k). These results suggest that differences in *SD1* and *GNP1* function are caused by divergent expression patterns, although our analyses indicate that both genes were expressed in the inflorescence meristem, albeit at different levels (Figure S7; Wu *et al*., 2016). A finer approach, such as single‐cell transcriptomics, will be required to unravel whether spatiotemporal differences in gene expression are responsible for different effects of these genes on panicle development. Finally, functional differences may be caused by different catalytic activities or products, such as AtGA20ox5 and other AtGA20oxs as the former only catalyses conversion of GA_12_ to GA_24_ (Plackett et al., 2012). Previous research suggests that GNP1/OsGA20ox1 may have higher affinities for GA_53_ than for GA_12_ (GA_1_ pathway), while SD1/OsGA20ox2 has higher affinities for GA_12_ than for GA_53_ (GA_4_ pathway; Kuroha *et al*., 2018; Toyomasu *et al*., 1997). Slightly different activities may explain the dose effects of GA in single mutants and double mutants involved in panicle development (Figure 4a–e).

### SLR1–KNOX class 1 protein interaction may mediate GA‐regulated panicle development

Previous studies have shown that KNOX class 1 proteins repressed the abundance of GA by promoting expression of GA metabolic genes or repressing expression of GA synthetic genes (Bolduc and Hake, 2009; Jasinski *et al*., 2005). However, *GNP1/OsGA20ox1* has also been shown to induce *KNOX* class 1 gene expression to regulate panicle meristem activity (Wu *et al*., 2016). It is still uncertain on how KNOX class 1 proteins participate in GA‐regulated panicle development in rice. In our study, *SD1* transcripts were detected in the branch primordium (Figure 3), which was overlapping with *KNOX* class 1 genes (i.e. OSH1, OSH15 and OSH6) (Harrop *et al*., 2016). This expression pattern indicates that *SD1* plays an essential role in regulating panicle development and may have some connection with *KNOX* class 1 genes.

DELLA proteins usually act as negative regulators in the GA signalling pathway (Zentella *et al*., 2007), and participate in GA‐induced plant growth and development by interaction several key proteins (Chen *et al*., 2017; Daviere and Achard, 2016; Huang *et al*., 2015). Although *SD1* has reported to improve rice yield by DELLA‐GRF4 interaction to regulate nitrogen and carbon metabolism (Li *et al*., 2018), the specific mechanisms by how GA modulates panicle development are still poorly understood. In our *sd1* mutants, SLR1 was accumulated in young inflorescence (Figure 6a), which may indicate its potential role on panicle development. Meanwhile, SLR1 was found to directly interact with KNOX class 1 proteins OSH1, OSH6, OSH15, OSH43 and OSH71, in our Y2H, BiFC and split‐luciferase assays (Figure 6; Figure S12). The N‐terminal domain of DELLA proteins is required for GA signal recognition by interaction with GA receptor GID1 protein (Itoh *et al*., 2002; Ueguchi‐Tanaka *et al*., 2007; Willige *et al*., 2007), and the C‐terminal domain is used to interact with other proteins to participate in plant development or target the DELLA for degradation (Dill *et al*., 2001; Dill *et al*., 2004; Gomi *et al*., 2004; Li *et al*., 2016; Van De Velde *et al*., 2017). Protein domain analysis revealed that the KNOX 1 and HOX domains of OSH1 could interact with the C‐terminal GRAS domain of SLR1 (Figure 6); interaction of the KNOX 1 domain with SLR1 impacted the ability of OSH1 to bind to promoters of downstream genes, known to be crucial for SAM formation and maintenance (Figure 7; Nagasaki *et al*., 2001; Tsuda *et al*., 2011).

We have detected expression of panicle‐related genes in the *sd1* mutant, and observed several genes were reduced distinctly such as *IPA1*, but others have no obvious change such as *TAW1* (Figure 5; Figure S11). According to public Chip‐seq data (Tsuda *et al*., 2014), OSH1 might not directly bind to *IPA1*, so we speculated that SLR1‐OSH1 interaction would not affect *IPA1* expression. It is likely that other *KNOX* class 1 proteins or other SLR interaction proteins regulate *IPA1* expression, which remains to be investigated.

Based on these findings, we have proposed a model for GA regulation of panicle development in *sd1* mutants and wild‐type plants. During the panicle development, in wild type DELLA (SLR1) is degraded and *KNOX* class 1 gene expression is increased, thus promoting the expression of OSH1‐activated downstream effector genes such as *ASP1* to promote panicle development. Conversely, in *sd1* mutants, non‐functional *SD1* leads to SLR1 accumulation and sequestration of functional OSH1 to reduce activation of genes involved in panicle development (Figure 7e).

In this study, the mechanisms on how *SD1* regulates panicle development are further elucidated, and thus provide a basis for high‐yield rice breeding. In addition, combining these two QTLs (*GNP1* and *SD1*) provides new breeding targets for panicle architecture refinement.

## Experimental procedures

### Plant materials and growth conditions

Chromosome segment substitution lines (CSSLs) bred from a *O. sativa* var. Nipponbare (japonica) × 9311 (indica) cross were received from Yang Zhou University (Zhang *et al*., 2011). All transgenic plants in this paper were sown during late spring and grown during summer in paddy fields at Shanghai (31.03°N, 121.45°E), China, during 2015–2020.

For QTL mapping, CSSL‐9 plants were back‐crossed to Nipponbare to generate the F_2_ mapping population, grown in Hainan province (18.1°N, 109.3°E). Molecular markers were designed according to sequence differences between the parent lines (Nipponbare and 9311) in the Rice SNP‐Seek Database (Mansueto *et al*., 2017).

### Construction of transgenic lines

For complementation, a whole genomic fragment of *SD1*, containing 2.7 kb coding sequences, 2.0 kb upstream promoter region and 0.7 kb downstream region, was amplified from Nipponbare genomic DNA, cloned into pCAMBIA1301 (In‐Fusion HD Cloning Kit; TaKaRa), and the resulting *pSD1:SD1* were transformed into *Agrobacterium tumefaciens* EHA105, and infiltrated into CSSL‐9 calli, as previously described (Li *et al*., 2006). The *pGNP1:GNP1* construct, containing a *GNP1* 3.0 kb promoter region and 1.1 kb coding sequence in pCAMBIA1301, was similarly generated and infiltrated into CSSL‐9 calli. The *pSD1:GNP1* construct contained the 3.0 kb *SD1* promoter upstream of the 1.1 kb *GNP1* coding sequence in pCAMBIA1301, again infiltrated into CSSL‐9 calli. All primers are listed in Table S1. To generate *p35S:SD1^Nip^* and *p35S:SD1^9311^* overexpression constructs, a full‐length coding sequence (open reading frame) of *SD1* was amplified from each parent (*SDI^Nip^* and *SD1^9311^*) using cDNA from rice primordia (see below), and cloned into pCAMBIA1301 under control of the 35S promoter (Table S1). To generate the *SD1* knockout lines, a sequence from the first exon (106–127 bp; TGAGGATGGAGCCCAAGATCC) was amplified with primers and cloned into pBIN‐sgR‐Cas9‐OsU3 vector (Table S1) (Biswas *et al*., 2020). To generate the *GPN1* knockout lines, two target sequences from the exon (24–43 bp, GCAGGAGGTGGTGTTCGACG; 347–366 bp, GCTACGCCAGCAGCTTCACG) were amplified with primers and cloned into pRGEB32 vector for CRISPR/Cas9‐mediated mutation (Table S1) (Xie *et al*., 2015). The homozygous mutants were obtained by segregation and sequencing of the T_1_ generation. To generate GFP expression constructs, the 2.0 kb promoter region from Nipponbare was amplified and cloned into a pCAMBIA1301:GFP vector containing the enhanced GFP (eGFP) coding sequence (He *et al*., 2016; Table S1). Primary and secondary branch primordia (panicle length was less than 1mm) were collected from transformed Nipponbare plants for GFP analysis. CSSL‐9, Nipponbare (Nip), and Kasalath were used as the recipients for *Agrobacterium*‐mediated transformation to generate the transgenic rice.

### Expression analysis

For quantitative reverse‐transcription PCR (qRT‐PCR), total RNA from seedling leaves, mature leaves, root, panicle and leaf sheath was extracted from rice tissues with TRIzol reagent (Invitrogen). For each sample, 1 μg of RNA was used to synthesize cDNA using the PrimeScriptRT reagent kit with gDNA eraser (TaKaRa), according to the manufacturer’s instructions. qRT‐PCR was performed using SYBR Premix Ex Taq (TaKaRa), according to the manufacturer’s instructions with the Bio‐Rad Real‐Time PCR System. Rice actin gene was used as an internal control to normalize the data. Measurements were obtained via the relative quantification method. Each experiment was repeated with three independent biological samples and three technical replicates. Primers of *OsGA2oxs* were referred to the study of Wu *et al*., (2016). Other primers are listed in Table S1.

For *in situ* hybridization, rice young panicle samples (<1mm) were fixed in FAA solution (10:50:5 formaldehyde: ethanol: acetic acid in water) for 24 h at 4 °C, then dehydrated with a graded ethanol solution, and embedded in paraffin according to Li *et al*., (2006). 8‐μm‐thick sections were cut using a Leica microtome (RM2235). Deparaffinization, probe hybridization and immunological detection of digoxigenin were performed as previously described (Kouchi and Hata, 1993). All probes in this paper were expressed under the T7 promoter using the DIG RNA labelling kit (Roche). For subsequent cloning of gene coding sequences, cDNA was generated from RNA collected from young panicle tissues.

### Yeast two‐hybrid assays

The full‐length and truncated coding sequences of *SLR1* from Nipponbare were amplified and cloned into *EcoR*I and *BamH*I restriction sites of *pGADT7* (TaKaRa), which encodes the GAL4 activation domain. The coding sequence of *KNOX* genes (*OSH1*, *OSH6*, *OSH15*, *OSH43* and *OSH71*) and truncated *OSH1* were amplified and cloned into *EcoR*I and *BamH*I restriction sites of *pGBKT7* (TaKaRa), which encodes the GAL4 binding domain. To detect protein–protein interactions, recombinant *pGBKT7‐KNOX* class 1 and *pGADT7‐SLR1* plasmids were co‐transformed into *Saccharomyces cerevisiae* strain AH109, according to the manufacturer’s instructions (TaKaRa). Transformants were selected on SD medium lacking SD/‐Trp/‐Leu/‐His/‐Ade.

### Bimolecular fluorescence complementation assays

The full‐length coding sequence of *SLR1* and *KNOX* class 1 were amplified and cloned into *pXY104‐cYFP* and *pXY106‐nYFP* plasmids, respectively. *SLR1‐cYFP* and *KNOX 1‐nYFP* plasmids were introduced into *A. tumefaciens* GV3101, which were grown in LB medium with 50 µg/mL kanamycin and 25 µg/mL rifampicin, resuspended in infection solution (10 mm MES and 200 μm acetosyringone) and co‐infiltrated into 3‐week‐old *N. benthamiana* leaves. The BiFC assay was performed as previously described (Zhang *et al*., 2020). After 48‐h incubation, fluorescent eYFP signals were detected at excitation 514 nm and emission 522–555 nm using a Leica SP8 confocal microscope.

### Yeast one‐hybrid assays

The full‐length coding sequence of *OSH1* was amplified and cloned into pB42AD vector (Ma *et al*., 2018). The *ASP1* promoter was cloned into pLacZ vector (Ma *et al*., 2018). The recombinant pB42AD‐OSH1 and pLacZ‐*ASP1* plasmids were co‐transformed into yeast strain EGY48 according to the Clontech transformation procedure. Transformants were grown on the medium lacking Ura/Trp and tested on SD/‐Trp/‐Ura plates with X‐gal, as described as Ma *et al*., (2018).

### Western blot analysis

Young panicles (<1 mm) from wild‐type and *sd1* plants were collected and ground into powder in liquid nitrogen. Proteins were extracted with TRIzol reagent (Invitrogen) based on Joy *et al*., (2018). The extraction protein was kept in 0.3 m guanidine hydrochloride. Proteins were separated on an SDS‐PAGE gel and immunodetected with anti‐SLR1 antibody (kindly provided by Professor Donglei Yang from Nanjing Agricultural University, 1:5000 dilution) and then anti‐tubulin antibody (Sigma, 1:5000 dilution).

### Luciferase assays

For dual‐luciferase assays, the 2.0 kb *SD1* promoter region from Nipponbare and 9311 was amplified and cloned into *pGREENII 0800* vector. For luciferase reporter assays, the effector was generated by inserting the coding sequence of *OSH1* into *pGREEN 000* plasmid (kindly provided by Hao Yu, National University of Singapore, Singapore) under control of the 35S promoter. The reporter was generated by inserting the 3.0‐kb promoter sequence of *ASP1* upstream of the *LUC* reporter gene in *pGREENII 0800* vector (provided by Hao Yu; see Table S1 for primers).

For split‐luciferase assays, the coding sequences of *KNOX* class 1 genes from Nipponbare were cloned into the pCAMBIA 1300‐nLuc plasmid, which encodes the N‐terminal luciferase domain. The coding sequence of *SLR1* from Nipponbare was cloned into pCAMBIA 1300‐cLuc plasmids, which encodes the C‐terminal luciferase domain (see Table S1 for primers).

All constructs above were introduced into tobacco leaves via *Agrobacterium*‐mediated transformation as described above. After 36‐h incubation in the dark, pictures were captured by a cooling CCD imaging apparatus (Tanon 5200). Tobacco leaves were ground on liquid nitrogen, and luciferase and Renilla activities were measured with a dual‐luciferase reporter assay kit (Promega), according to the manufacturer’s instructions.

### Subcellular localization assays

The coding regions of *SD1* and *GNP1* were cloned into pCAMBIA1301‐eGFP. We used 12‐day‐old rice leaves to generate protoplasts, and transform them with vectors as described by Bart *et al*., (2006). Fluorescence was analysed by a Leica SP8 confocal microscope (Leica TCS SP8 X) at excitation 488–507 nm for monitor eGFP signal, and 597–648 nm for mCherry of endoplasmic reticulum marker.

### Phylogenetic analysis

Homologous and paralogous amino acid sequences of SD1 from different species were obtained from National Center for Biotechnology Information (NCBI, https://www.ncbi.nlm.nih.gov/), and aligned with MEGA 5 (Tamura *et al*., 2011). The aligned sequences were used to construct a phylogenetic with neighbour‐joining method using MEGA 5 with the following parameters: Poisson correction, pairwise deletion and 1000 bootstrap replicates.

### Quantification of endogenous GA

For GA quantification, young inflorescences (<1 cm) of *sd1* mutants (*sd1‐1*) and Nipponbare plants were harvested into liquid nitrogen. Approximately 500 mg inflorescences were collected for each of three biological samples. GAs were measured by an ultra‐performance liquid chromatography–mass spectroscopy (UPLC‐MS) according to Xin *et al*., (2020).

### Statistical analysis

Data analysis was carried out by Student’s *t*‐test of Microsoft Excel software.

## Competing interests

The authors declare no competing interests and approved the paper.

## Author contributions

D. Z. directed project. S. S. performed the experiments. D. Z., J. H., S. S. and W. L. conceived and designed the research. X. C. and M. C. provided the transgenic technology. Z. L. conducted the fieldwork. S. C. and S. B. participated in the experiments. C. Z. and Q. L. created CSSL materials. S. S., J. H. and D. Z. wrote the paper.

## Supporting information


**Figure S1**. Plant height of 136 CSSLs plus 2 parent lines grown at Shanghai in 2015. The red arrows show the position of CSSL‐9, Nipponbare (Nip), and 9311.

**Figure S2**. Alignment of coding sequences of *SD1^9311^* and *SD1^Kas^*. Two SNPs at positions +654 bp and +1026 bp are boxed in red. Nucleotides at +299 bp and +1019 bp (blue boxes) that had mis‐sense SNPs between *SD1^9311 and^ SD1^Nip^* were the same in *SD1^9311^* and *SD1^Kas^*.

**Figure S3**. Transcriptional activity of different *SD1* promoter variants *in vivo* and *in vitro*. (a) *SD1* expression in young panicle in CSSL‐9 and Nipponbare. Mean ± SE, *n* = 3. (b) LUC/REN ratio of different *SD1* promoter variants used in dual‐luciferase reporter assays in *N. benthamiana*. Mean ± SE, *n* = 3. Differences in wild‐type plant indicated **P < 0.01, *t*‐test.

**Figure S4**. Knock out of *SD1* in cultivar Kasalath. (a) Plant and panicle architecture of of *sd1* mutants in the Kasalath background. Bar = 20 cm. (b) Target sequence of CRISPR/Cas9‐mediated *sd1^Kas^* knockout line. (c–g) Agronomic and panicle traits of wild type Kasalath (Kas) and *sd1* plants, showing (c) plant height; (d) panicle length, number of (e) primary and (f) secondary branches per panicle; and (g) number of grains in the main panicle. Mean ± SE, *n* = 20. Difference to wild type indicated: **P < 0.01, t‐test.

**Figure S5**. Phylogenetic tree of GA_20_ oxidase proteins in six species. Os, *Oryza sativa*; At, *Arabidopsis thaliana*; Ta, *Triticum aestivum*; Hv, *Hordeum vulgare*; Sb, *Sorghum bicolor*; and Zm, *Zea mays*.

**Figure S6**. Localization of GNP1 and SD1 proteins in rice protoplasts. *SD1‐eGFP* and *GNP1‐eGFP* were driven by the 35S promoter. Bar = 5 μm. mCherry is the endoplasmic reticulum marker.

**Figure S7**. *SD1* and *GNP1* expression levels during rice growth. ml, mature leaf; yl, young leaf; yl sh, young leaf sheath; pbp, primary branch primordium; sbp, secondary branch primordium; el, elongated stem; pl, panicle length. Mean ± SE, *n* = 3. Differences between tissue pairs indicated: **P < 0.01, *t*‐test.

**Figure S8**. *GNP1* expression level under *pGNP1:GNP1* transgenic plants. Mean ± SE, *n* = 3. Differences between tissue pairs indicated: **P < 0.01, *t*‐test.

**Figure S9**. Content of two bioactive GAs in young *sd1* and wild type (Nipponbare) inflorescences. Mean ± SE, *n* = 3. No differences were observed.

**Figure S10**. Expression of GA catabolic enzymes in Nipponbare (Nip) and *sd1* mutants. Mean ± SE, *n* = 3. Differences between tissue pairs indicated: *P < 0.05, **P < 0.01, *t*‐test.

**Figure S11**. Relative expression of genes involved in panicle development in wild type and *sd1* panicle branch primordia. Mean ± SE, *n* = 3. No differences were observed.

**Figure S12**. Other KNOX class 1 proteins can directly interact with SLR1. (a) KNOX class 1 proteins interact with SLR1 in Y2H assays. KNOX class 1 proteins were fused to the GAL4 binding domain (BD); SLR1 was fused to the GAL4 activation domain (AD). (b) KNOX class 1 proteins interact with SLR1 in a BiFC assay. KNOX class 1 proteins were fused to nYFP; SLR1 was fused to cYFP. nYFP, N‐terminal yellow fluorescent protein; cYFP, C‐terminal yellow fluorescent protein. (c) Split‐luciferase assays between KNOX class 1 proteins and SLR1 with controls in tobacco leaves. cLuc, C‐terminal luciferase; nLuc, N‐terminal luciferase.

**Figure S13**. Relative expression of *KNOX* class 1 genes involved in panicle development in wild type and *sd1* panicle branch primordia. Mean ± SE, *n* = 3. Differences to wild type plants indicated: *P < 0.05, **P < 0.01, *t*‐test.

**Table S1**. Primers used in this study.

**Table S2**. Variation of 3K varieties at genomic position of 38 385 064 bp (encoding position at 1026 bp).
