# Supplementary material for: Gibberellins orchestrate panicle architecture mediated by DELLA–KNOX signalling in rice
Source: Plant Biotechnol J. 2021 Aug 24;19(11):2304–18. doi: 10.1111/pbi.13661 (PMC8541776; doi:10.1111/pbi.13661)
Supplement: Supplementary file 1 — Figure S1. Plant height of 136 CSSLs plus 2 parent lines grown at Shanghai in 2015. The red arrows show the position of CSSL‐9, Nipponbare (Nip), and 9311. [file PBI-19-2304-s011.pptx]

## Slide 1
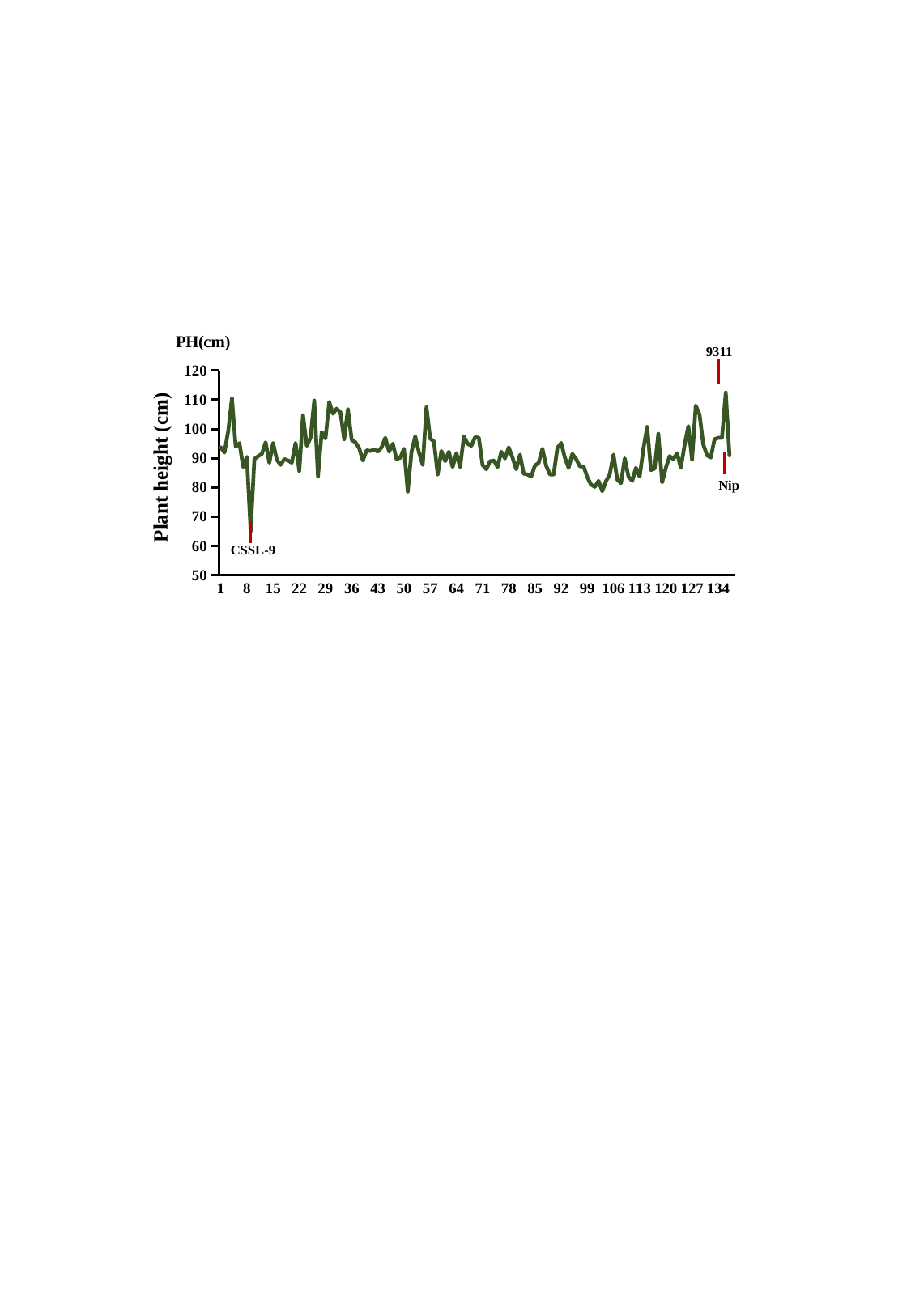

### Chart: PH(cm)
| Category | 平均值 |
|---|---|9311
Plant height (cm)
Nip
CSSL-9
Supplementary data 3. SD1 expression between CSSL-9 and Nipponbare.
