# Supplementary figures and images for: Gibberellins orchestrate panicle architecture mediated by DELLA–KNOX signalling in rice
Source: Plant Biotechnol J. 2021 Aug 24;19(11):2304–18. doi: 10.1111/pbi.13661 (PMC8541776; doi:10.1111/pbi.13661)

## Slide 1
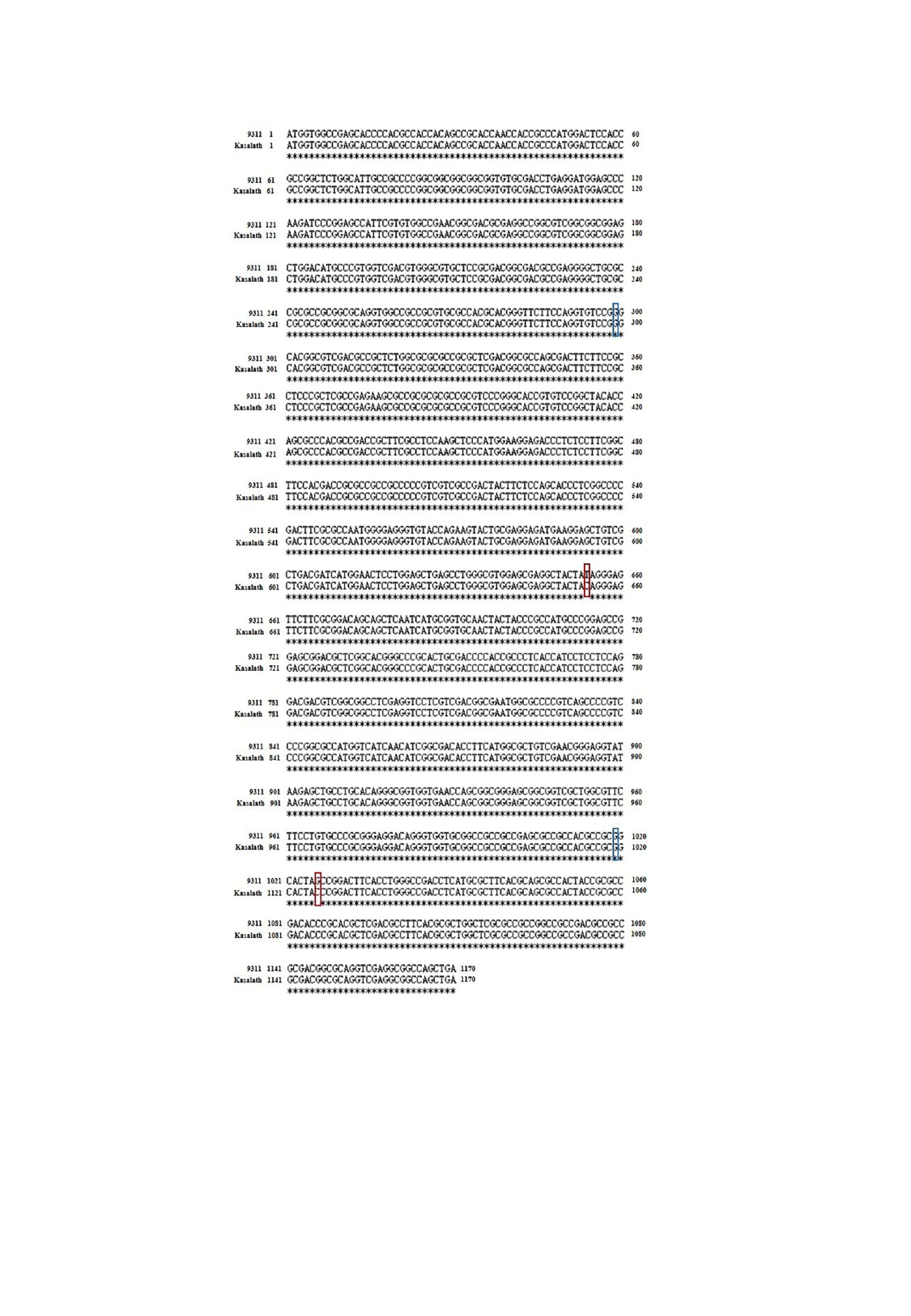

Supplement: Supplementary file 2 — Figure S2. Alignment of coding sequences of SD19311 and SD1Kas . Two SNPs at positions +654 bp and +1026 bp are boxed in red. Nucleotides at +299 bp and +1019 bp (blue boxes) that had mis‐sense SNPs between SD19311 and SD1Nip were the same in SD19311 and SD1Kas . [file PBI-19-2304-s010.pptx]

## Slide 1
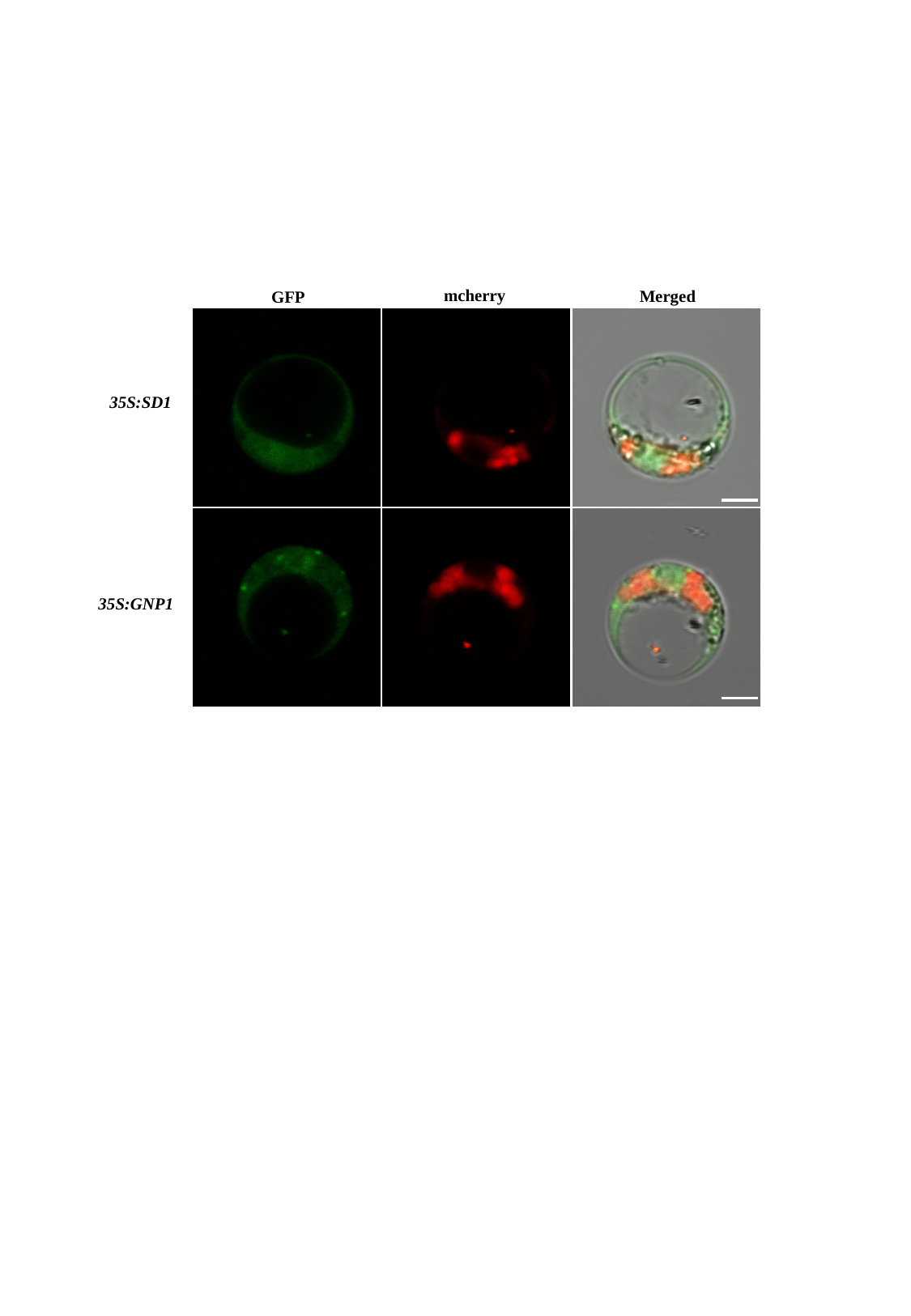

mcherry
Merged
GFP
35S:SD1
35S:GNP1

Supplement: Supplementary file 6 — Figure S6. Localization of GNP1 and SD1 proteins in rice protoplasts. SD1‐eGFP and GNP1‐eGFP were driven by the 35S promoter. Bar = 5 μm. mCherry is the endoplasmic reticulum marker. [file PBI-19-2304-s009.pptx]

## Slide 1
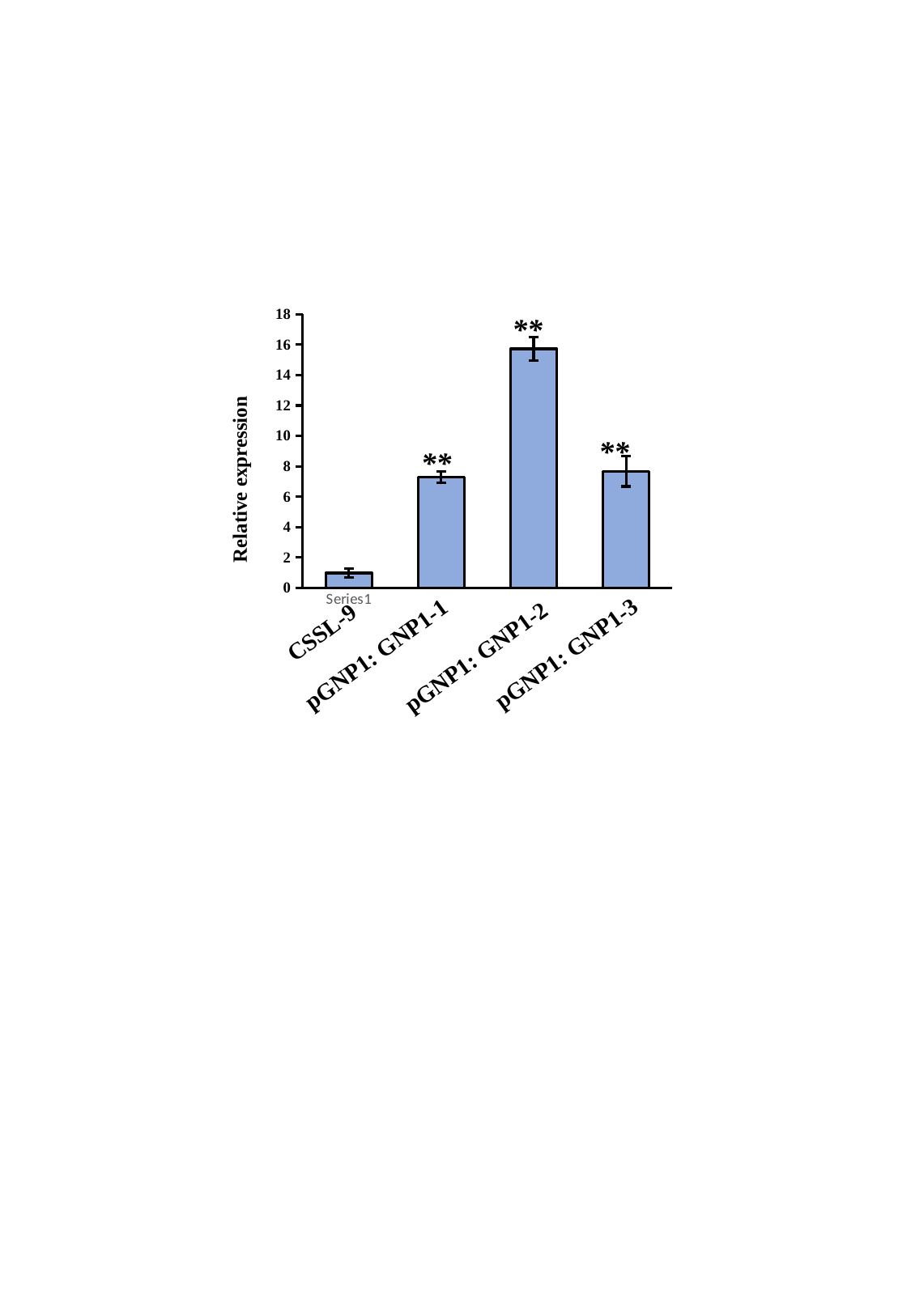

### Chart
| Category | |
|---|---|
| | 0.9824064479352064 |
| | 7.285236440050606 |
| | 15.725573706150863 |
| | 7.667934268307291 |CSSL-9
pGNP1: GNP1-1
pGNP1: GNP1-3
pGNP1: GNP1-2
Relative expression
**
**
**

Supplement: Supplementary file 8 — Figure S8. GNP1 expression level under pGNP1:GNP1 transgenic plants. Mean ± SE, n = 3. Differences between tissue pairs indicated: **P < 0.01, t‐test. [file PBI-19-2304-s004.pptx]

## Slide 1
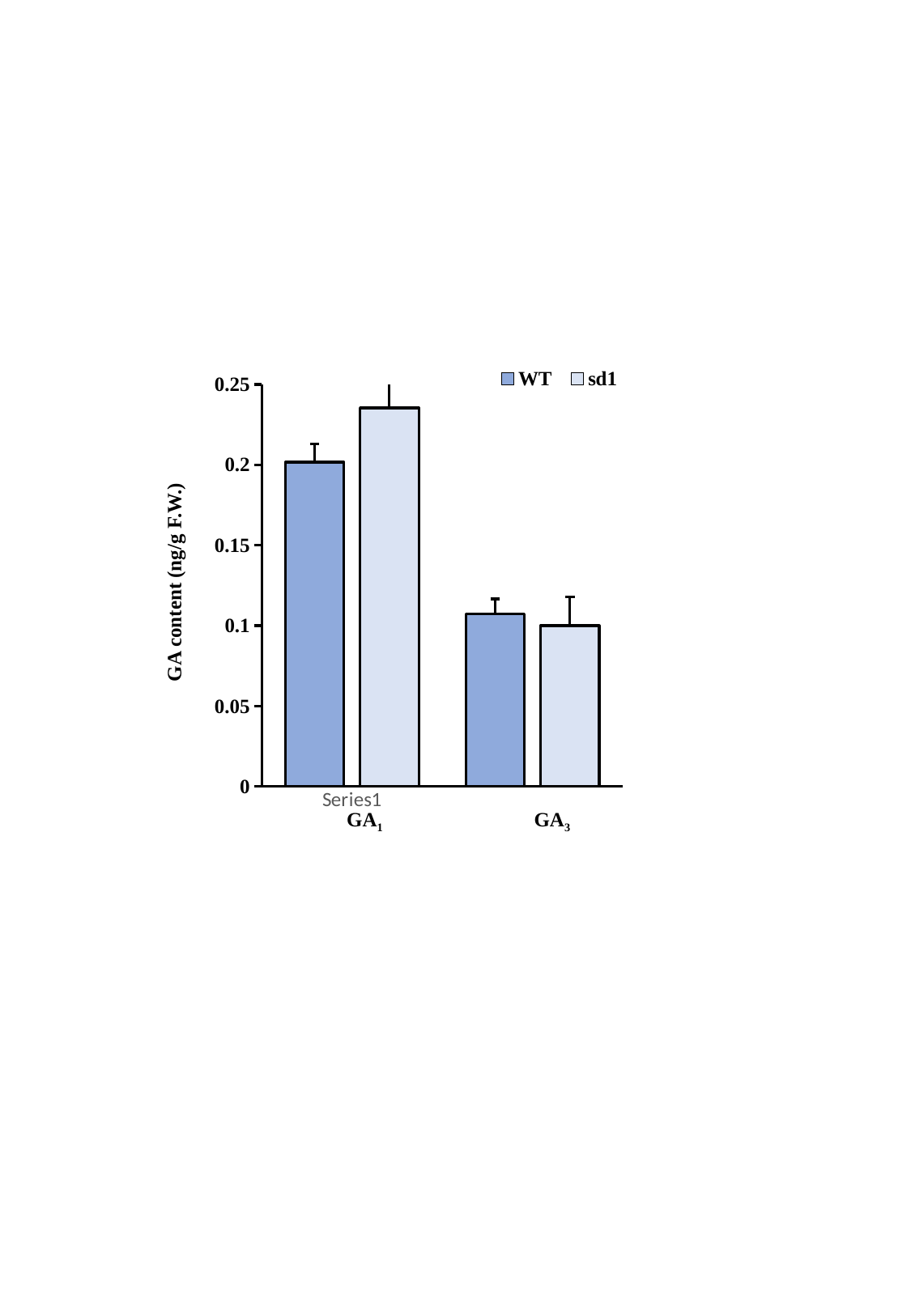

### Chart
| Category | WT | sd1 |
|---|---|---|
| | 0.20166666666666666 | 0.2353333333333333 |
| | 0.10733333333333332 | 0.09999999999999999 |GA1
GA3
GA content (ng/g F.W.)

Supplement: Supplementary file 9 — Figure S9. Content of two bioactive GAs in young sd1 and wild type (Nipponbare) inflorescences. Mean ± SE, n = 3. No differences were observed. [file PBI-19-2304-s003.pptx]
