# Supplementary material for: Gibberellins orchestrate panicle architecture mediated by DELLA–KNOX signalling in rice
Source: Plant Biotechnol J. 2021 Aug 24;19(11):2304–18. doi: 10.1111/pbi.13661 (PMC8541776; doi:10.1111/pbi.13661)
Supplement: Supplementary file 3 — Figure S3. Transcriptional activity of different SD1 promoter variants in vivo and in vitro. (a) SD1 expression in young panicle in CSSL‐9 and Nipponbare. Mean ± SE, n = 3. (b) LUC/REN ratio of different SD1 promoter variants used in dual‐luciferase reporter assays in N. benthamiana. Mean ± SE, n = 3. Differences in wild‐type plant indicated **P < 0.01, t‐test. [file PBI-19-2304-s006.pptx]

## Slide 1
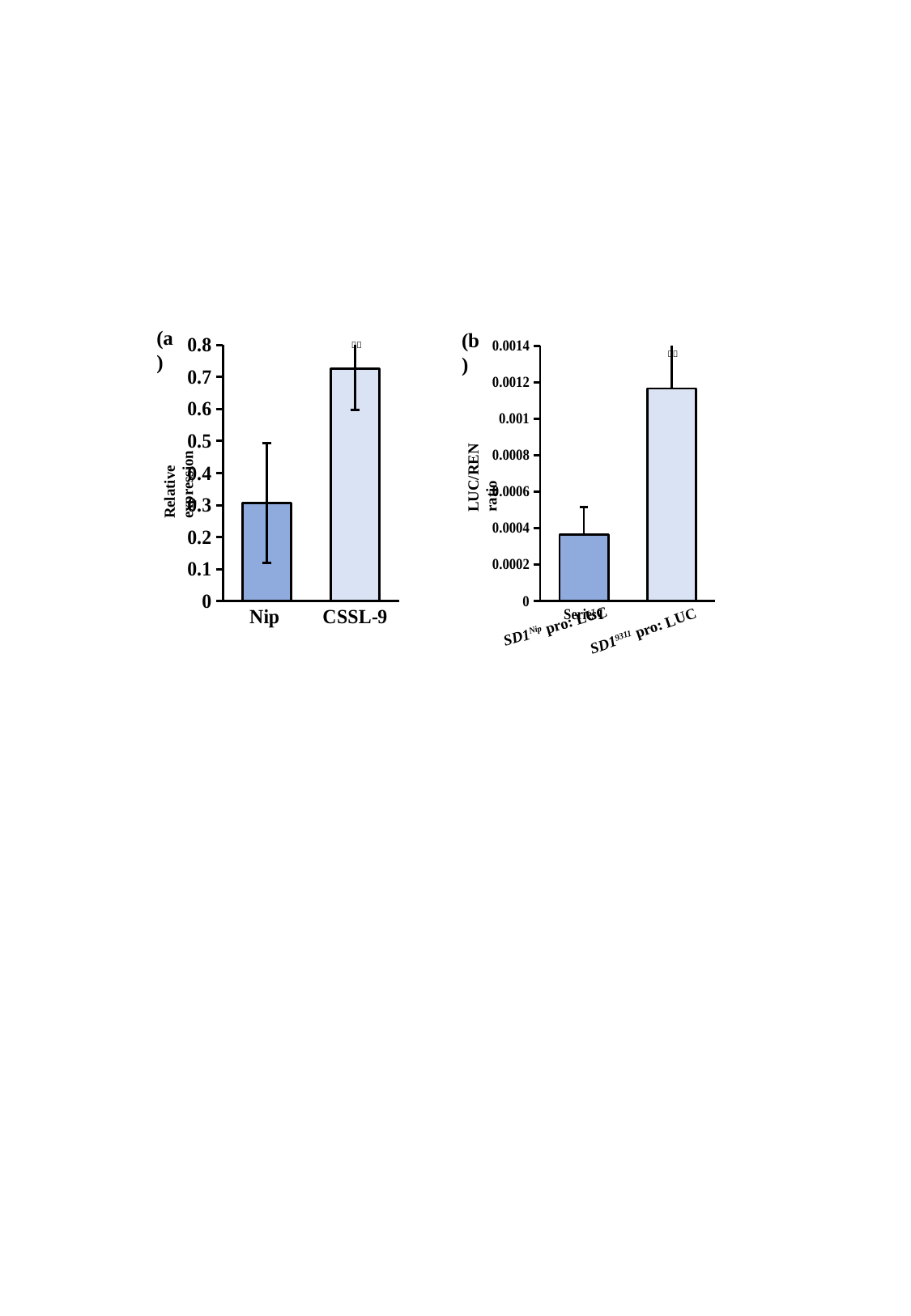

(a)
(b)
### Chart
| Category | 2-△△Ct |
|---|---|
| Nip | 0.3065748229026912 |
| CSSL-9 | 0.7270051273271646 |
### Chart
| Category | |
|---|---|
| | 0.00036666666666666667 |
| | 0.0011666666666666665 |＊＊
＊＊
Relative expression
LUC/REN ratio
SD1Nip pro: LUC
SD19311 pro: LUC
