# Supplementary material for: Gibberellins orchestrate panicle architecture mediated by DELLA–KNOX signalling in rice
Source: Plant Biotechnol J. 2021 Aug 24;19(11):2304–18. doi: 10.1111/pbi.13661 (PMC8541776; doi:10.1111/pbi.13661)
Supplement: Supplementary file 4 — Figure S4. Knock out of SD1 in cultivar Kasalath. (a) Plant and panicle architecture of of sd1 mutants in the Kasalath background. Bar = 20 cm. (b) Target sequence of CRISPR/Cas9‐mediated sd1Kas knockout line. (c–g) Agronomic and panicle traits of wild type Kasalath (Kas) and sd1 plants, showing (c) plant height; (d) panicle length, number of (e) primary and (f) secondary branches per panicle; and (g) number of grains in the main panicle. Mean ± SE, n = 20. Difference to wild type indicated: **P < 0.01, t‐test. [file PBI-19-2304-s015.pptx]

## Slide 1
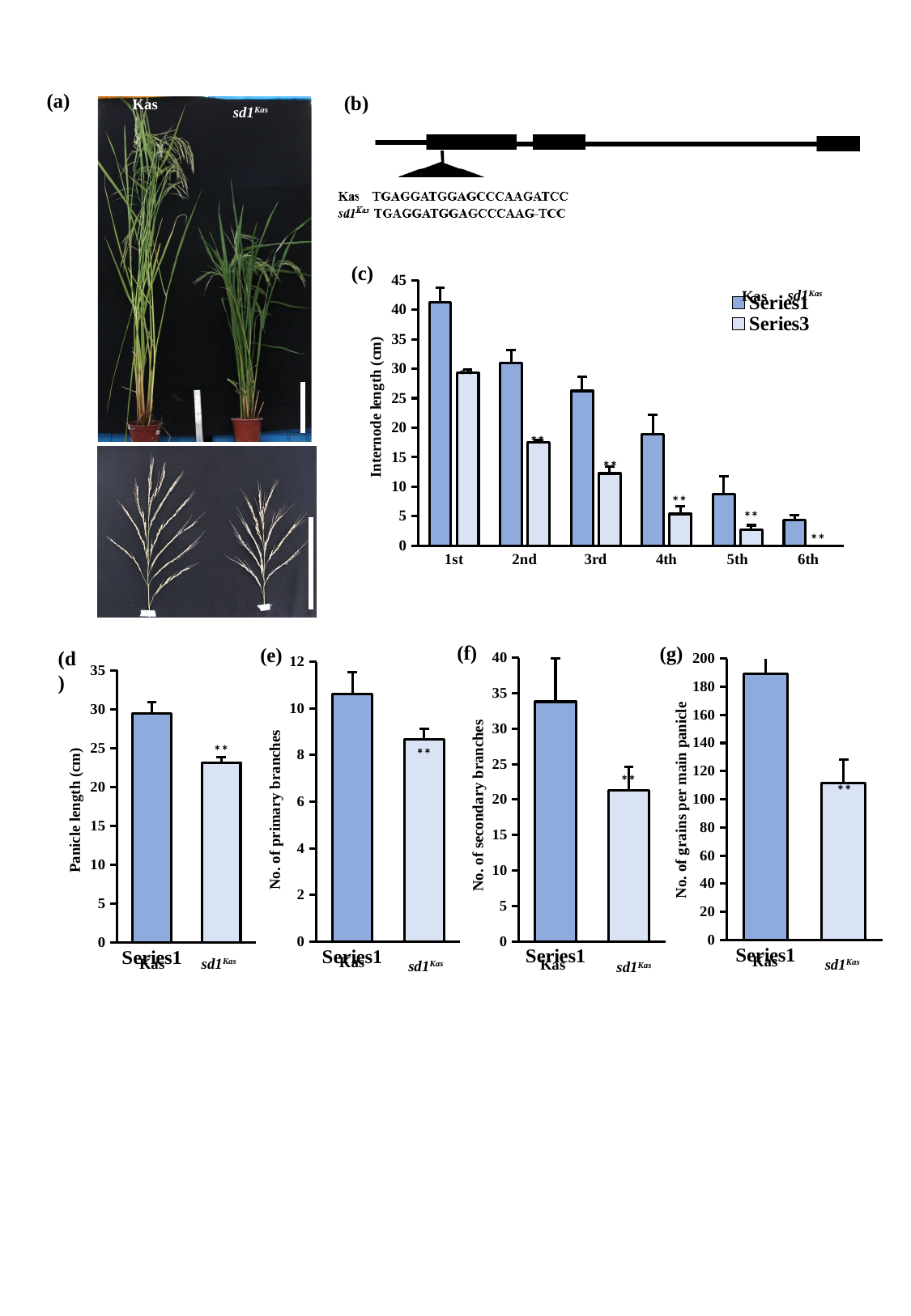

(a)
(b)
Kas
sd1Kas
### Chart
| Category | | |
|---|---|---|
| 1st | 41.321739130434786 | 29.28333333333333 |
| 2nd | 31.019047619047623 | 17.5 |
| 3rd | 26.23888888888889 | 12.233333333333334 |
| 4th | 18.830769230769228 | 5.366666666666667 |
| 5th | 8.754545454545454 | 2.625 |
| 6th | 4.325 | None |Kas
sd1Kas
Kas
(c)
**
**
**
**
**
**
(f)
(g)
### Chart
| Category | 总粒数 |
|---|---|
| | 189.1875 |
| | 111.66666666666667 |Kas
sd1Kas
No. of grains per main panicle
(e)
(d)
### Chart
| Category | 二次枝梗数 |
|---|---|
| | 33.8125 |
| | 21.333333333333332 |Kas
sd1Kas
No. of secondary branches
### Chart
| Category | PL |
|---|---|
| | 29.469565217391306 |
| | 23.150000000000002 |Kas
sd1Kas
Panicle length (cm)
### Chart
| Category | 一次枝梗数 |
|---|---|
| | 10.625 |
| | 8.666666666666666 |Kas
sd1Kas
No. of primary branches
**
**
**
**
