# Supplementary material for: Gibberellins orchestrate panicle architecture mediated by DELLA–KNOX signalling in rice
Source: Plant Biotechnol J. 2021 Aug 24;19(11):2304–18. doi: 10.1111/pbi.13661 (PMC8541776; doi:10.1111/pbi.13661)
Supplement: Supplementary file 5 — Figure S5. Phylogenetic tree of GA20 oxidase proteins in six species. Os, Oryza sativa; At, Arabidopsis thaliana; Ta, Triticum aestivum; Hv, Hordeum vulgare; Sb, Sorghum bicolor; and Zm, Zea mays. [file PBI-19-2304-s002.pptx]

## Slide 1
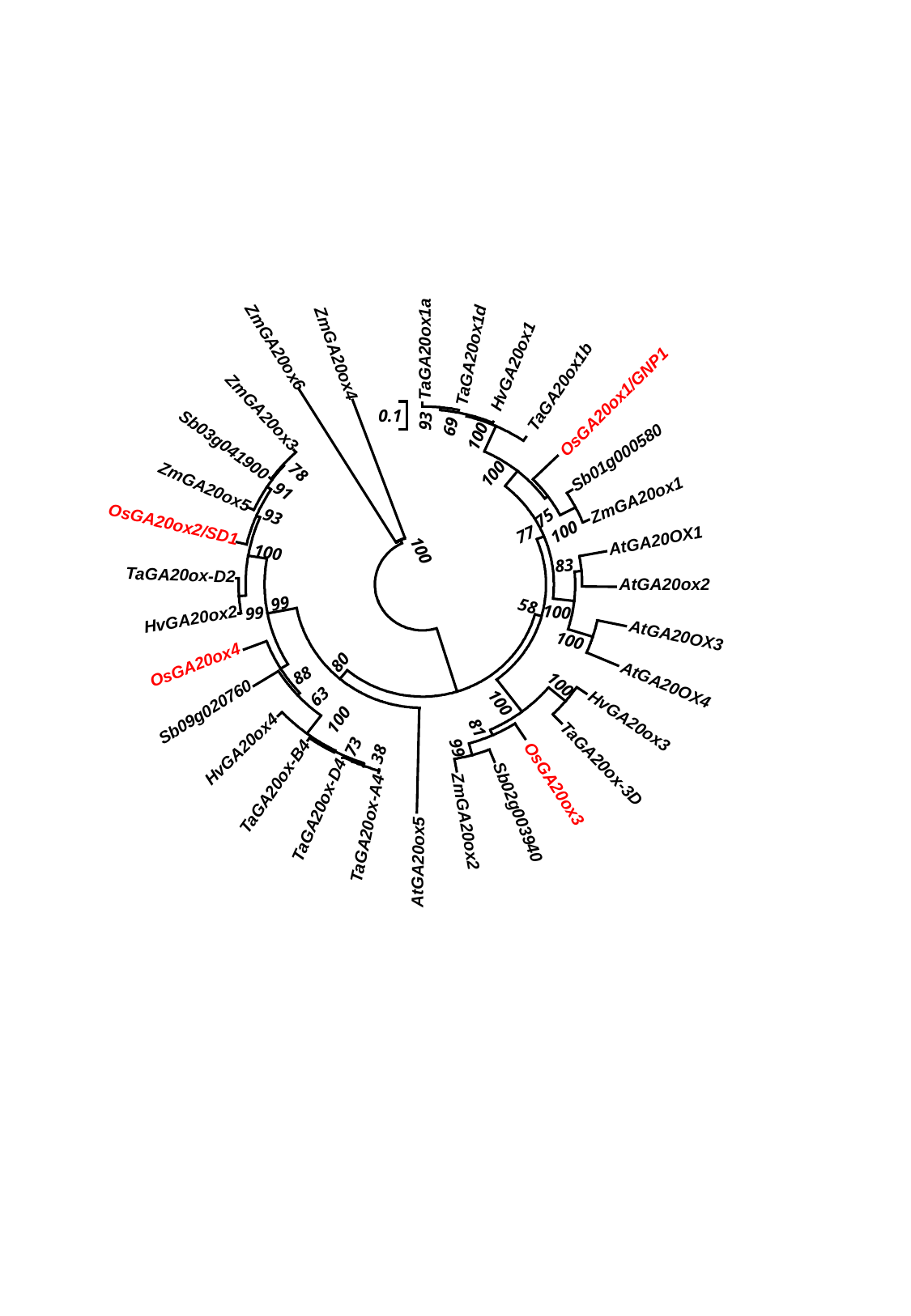

ZmGA20ox6
TaGA20ox1a
ZmGA20ox4
TaGA20ox1d
HvGA20ox1
TaGA20ox1b
OsGA20ox1/GNP1
ZmGA20ox3
0.1
93
69
100
Sb03g041900
Sb01g000580
78
100
ZmGA20ox5
91
ZmGA20ox1
93
75
OsGA20ox2/SD1
100
77
AtGA20OX1
100
100
83
TaGA20ox-D2
AtGA20ox2
99
58
100
99
HvGA20ox2
AtGA20OX3
100
80
OsGA20ox4
88
AtGA20OX4
100
63
100
Sb09g020760
100
HvGA20ox3
81
73
99
HvGA20ox4
38
TaGA20ox-3D
OsGA20ox3
TaGA20ox-B4
TaGA20ox-D4
Sb02g003940
ZmGA20ox2
TaGA20ox-A4
AtGA20ox5
