# Supplementary material for: Gibberellins orchestrate panicle architecture mediated by DELLA–KNOX signalling in rice
Source: Plant Biotechnol J. 2021 Aug 24;19(11):2304–18. doi: 10.1111/pbi.13661 (PMC8541776; doi:10.1111/pbi.13661)
Supplement: Supplementary file 7 — Figure S7. SD1 and GNP1 expression levels during rice growth. ml, mature leaf; yl, young leaf; yl sh, young leaf sheath; pbp, primary branch primordium; sbp, secondary branch primordium; el, elongated stem; pl, panicle length. Mean ± SE, n = 3. Differences between tissue pairs indicated: **P < 0.01, t‐test. [file PBI-19-2304-s001.pptx]

## Slide 1
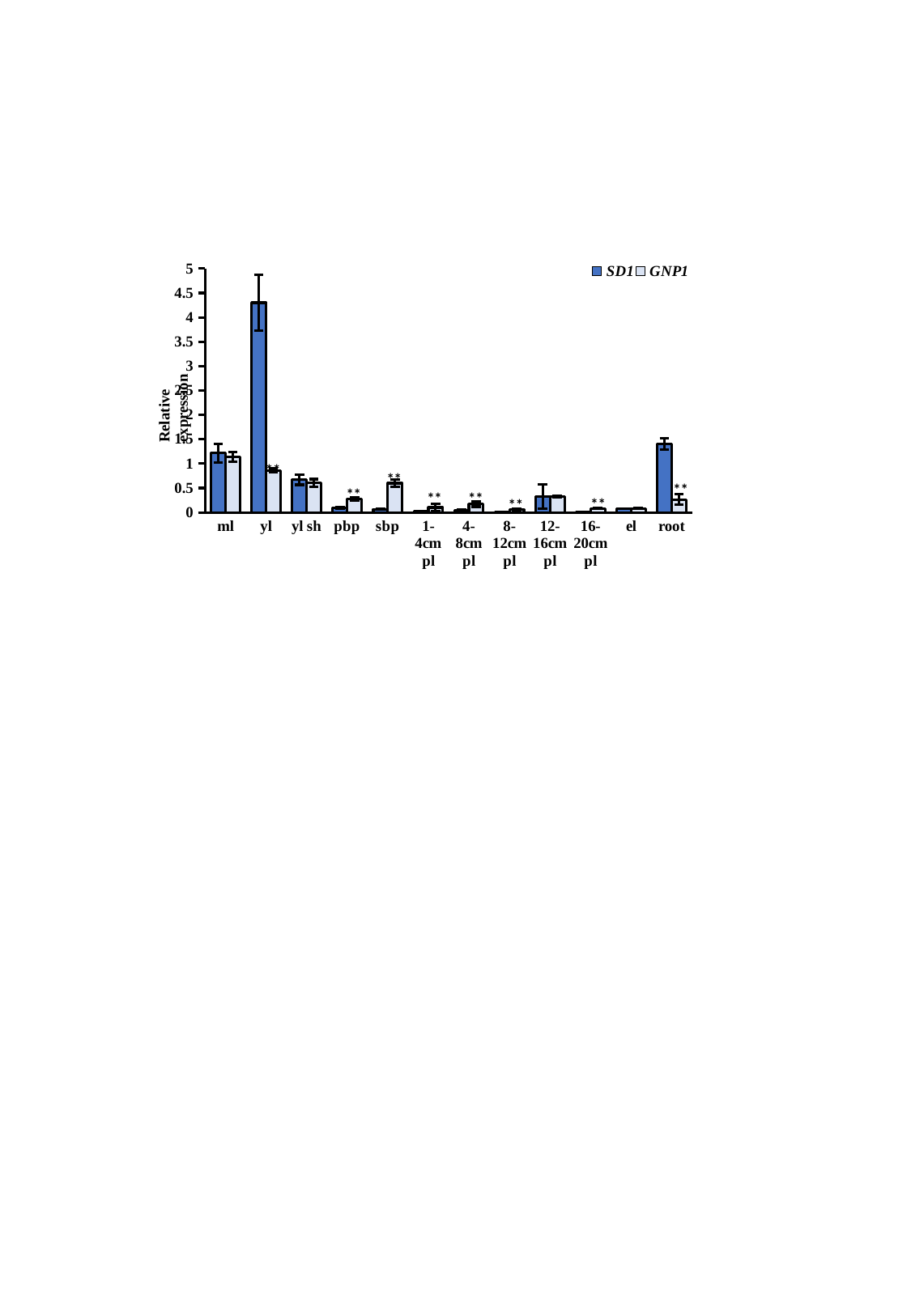

### Chart
| Category | SD1 | GNP1 |
|---|---|---|
| ml | 1.2150261191250529 | 1.1401171761158804 |
| yl | 4.298245988009175 | 0.8596620414787912 |
| yl sh | 0.6671531704529851 | 0.6024508826432862 |
| pbp | 0.09209428658540242 | 0.2735025073025343 |
| sbp | 0.06281778375714665 | 0.5984156000926905 |
| 1-4cm pl | 0.03113427810844868 | 0.09936769355826318 |
| 4-8cm pl | 0.040569722486871254 | 0.17403560935355986 |
| 8-12cm pl | 0.007622490975267529 | 0.0551803870878157 |
| 12-16cm pl | 0.3224331682819208 | 0.3234647497159481 |
| 16-20cm pl | 0.010012994190584995 | 0.07729618633216156 |
| el | 0.08004098761873858 | 0.08027878672788523 |
| root | 1.4056230957373523 | 0.26144481245606516 |Relative expression
**
**
**
**
**
**
**
**
