# Supplementary material for: Gibberellins orchestrate panicle architecture mediated by DELLA–KNOX signalling in rice
Source: Plant Biotechnol J. 2021 Aug 24;19(11):2304–18. doi: 10.1111/pbi.13661 (PMC8541776; doi:10.1111/pbi.13661)
Supplement: Supplementary file 10 — Figure S10. Expression of GA catabolic enzymes in Nipponbare (Nip) and sd1 mutants. Mean ± SE, n = 3. Differences between tissue pairs indicated: *P < 0.05, **P < 0.01, t‐test. [file PBI-19-2304-s014.pptx]

## Slide 1
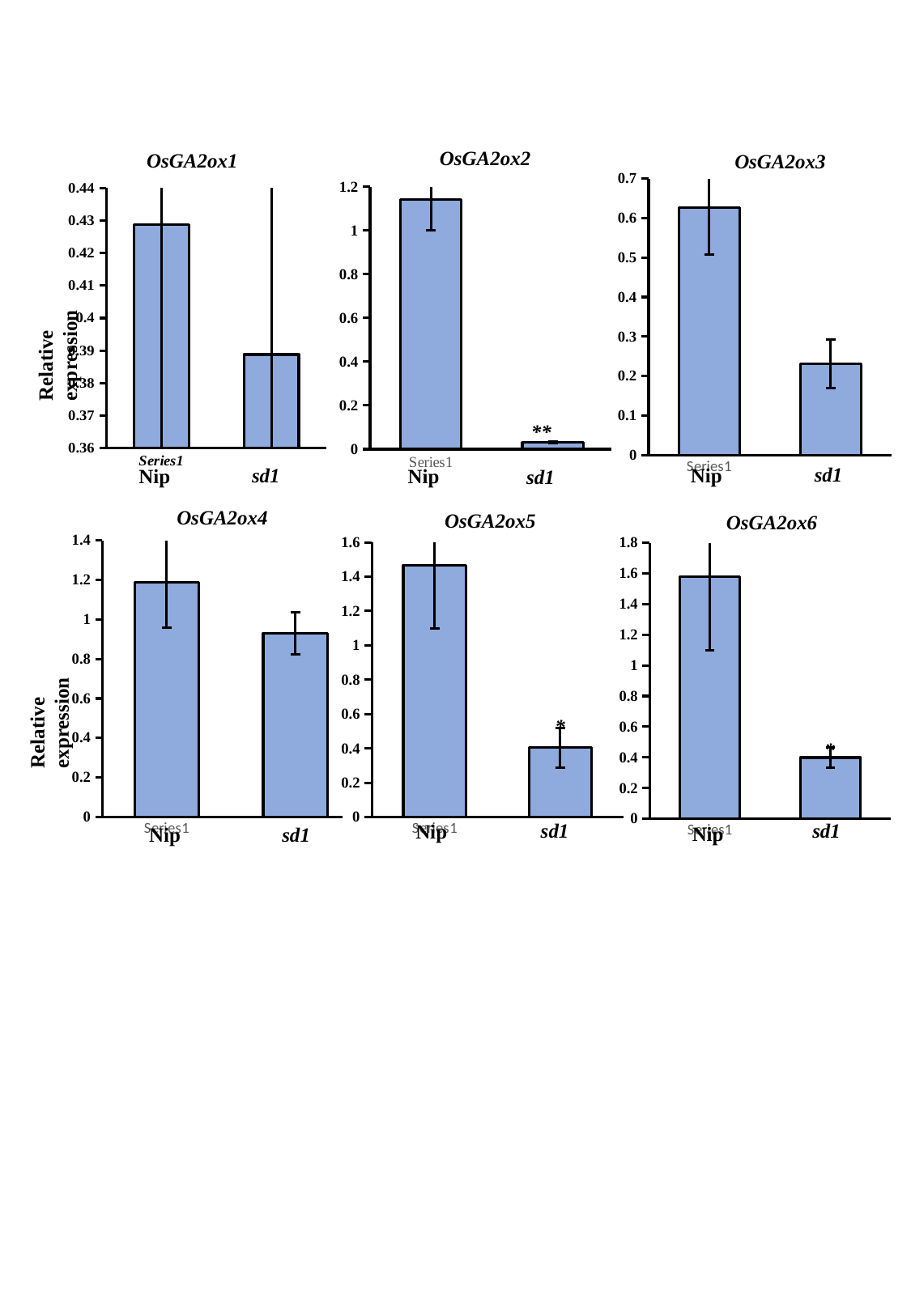

OsGA2ox2
### Chart
| Category | |
|---|---|
| | 1.1417129487814517 |
| | 0.030696335683094152 |Nip
sd1
OsGA2ox1
### Chart
| Category | |
|---|---|
| | 0.4287777916349055 |
| | 0.38877368728782113 |sd1
Nip
OsGA2ox3
### Chart
| Category | |
|---|---|
| | 0.6269459968864567 |
| | 0.23127486177300996 |sd1
Nip
Relative expression
**
OsGA2ox4
### Chart
| Category | |
|---|---|
| | 1.1895150608758036 |
| | 0.9296622348778268 |Nip
sd1
OsGA2ox5
### Chart
| Category | |
|---|---|
| | 1.465936328924845 |
| | 0.4034546979602745 |sd1
Nip
OsGA2ox6
### Chart
| Category | |
|---|---|
| | 1.5792282365044295 |
| | 0.3988879598208566 |sd1
Nip
Relative expression
*
*
