# Supplementary material for: Gibberellins orchestrate panicle architecture mediated by DELLA–KNOX signalling in rice
Source: Plant Biotechnol J. 2021 Aug 24;19(11):2304–18. doi: 10.1111/pbi.13661 (PMC8541776; doi:10.1111/pbi.13661)
Supplement: Supplementary file 11 — Figure S11. Relative expression of genes involved in panicle development in wild type and sd1 panicle branch primordia. Mean ± SE, n = 3. No differences were observed. [file PBI-19-2304-s013.pptx]

## Slide 1
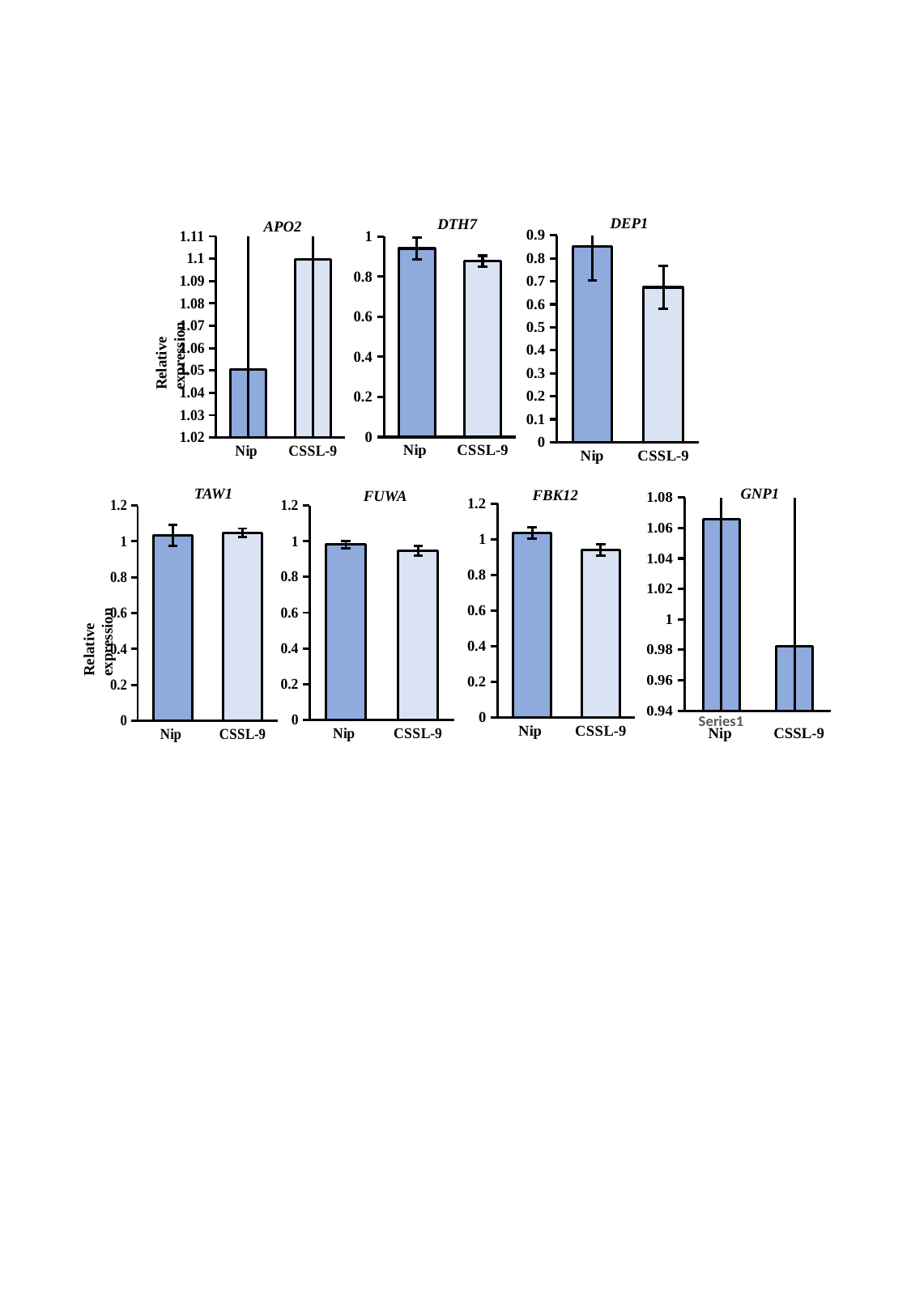

DEP1
DTH7
### Chart
| Category |
|---|APO2
### Chart
| Category | |
|---|---|
| Nip | 0.8511112189344994 |
| CSSL-9 | 0.6736815177127274 |
### Chart
| Category | |
|---|---|
| Nip | 1.0503338996802405 |
| CSSL-9 | 1.0996050192199671 |
### Chart
| Category | |
|---|---|
| Nip | 0.941062853780021 |
| CSSL-9 | 0.877039522287177 |Relative expression
TAW1
GNP1
### Chart
| Category | |
|---|---|
| | 1.0655823915825924 |
| | 0.9824064479352064 |Nip
CSSL-9
FBK12
FUWA
### Chart
| Category | |
|---|---|
| Nip | 1.0345066141885262 |
| CSSL-9 | 1.047583660962567 |
### Chart
| Category | |
|---|---|
| Nip | 1.0357806877201643 |
| CSSL-9 | 0.9400751730593601 |
### Chart
| Category | |
|---|---|
| Nip | 0.9819101644936571 |
| CSSL-9 | 0.9464364648473066 |Relative expression
