# Supplementary material for: Gibberellins orchestrate panicle architecture mediated by DELLA–KNOX signalling in rice
Source: Plant Biotechnol J. 2021 Aug 24;19(11):2304–18. doi: 10.1111/pbi.13661 (PMC8541776; doi:10.1111/pbi.13661)
Supplement: Supplementary file 12 — Figure S12. Other KNOX class 1 proteins can directly interact with SLR1. (a) KNOX class 1 proteins interact with SLR1 in Y2H assays. KNOX class 1 proteins were fused to the GAL4 binding domain (BD); SLR1 was fused to the GAL4 activation domain (AD). (b) KNOX class 1 proteins interact with SLR1 in a BiFC assay. KNOX class 1 proteins were fused to nYFP; SLR1 was fused to cYFP. nYFP, N‐terminal yellow fluorescent protein; cYFP, C‐terminal yellow fluorescent protein. (c) Split‐luciferase assays between KNOX class 1 proteins and SLR1 with controls in tobacco leaves. cLuc, C‐terminal luciferase; nLuc, N‐terminal luciferase. [file PBI-19-2304-s012.pptx]

## Slide 1
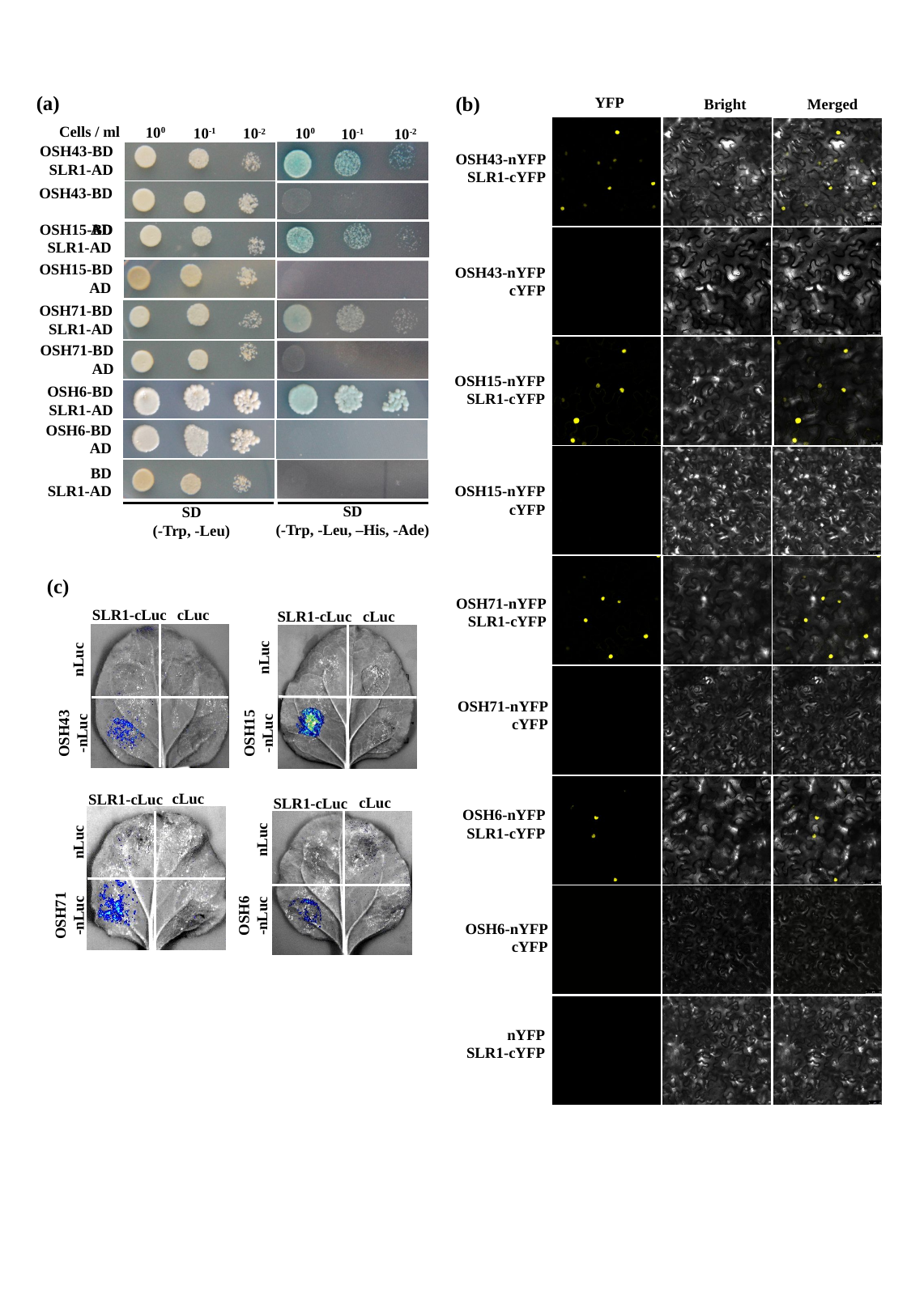

(a)
(b)
YFP
Bright
Merged
OSH43-nYFP
 SLR1-cYFP
OSH71-nYFP
SLR1-cYFP
OSH71-nYFP
cYFP
nYFP
SLR1-cYFP
OSH43-nYFP
cYFP
OSH15-nYFP
SLR1-cYFP
OSH6-nYFP
SLR1-cYFP
OSH6-nYFP
cYFP
OSH15-nYFP
cYFP
Cells / ml
100
10-1
100
10-2
10-1
10-2
OSH43-BD
SLR1-AD
OSH43-BD AD
OSH15-BD
 SLR1-AD
OSH15-BD
 AD
OSH71-BD
SLR1-AD
OSH71-BD
AD
OSH6-BD
SLR1-AD
OSH6-BD
AD
BD
SLR1-AD
SD
(-Trp, -Leu, –His, -Ade)
SD
(-Trp, -Leu)
(c)
SLR1-cLuc
cLuc
nLuc
OSH43-nLuc
cLuc
SLR1-cLuc
nLuc
OSH15-nLuc
cLuc
SLR1-cLuc
nLuc
OSH71-nLuc
cLuc
SLR1-cLuc
nLuc
OSH6
-nLuc
