# Supplementary material for: Gibberellins orchestrate panicle architecture mediated by DELLA–KNOX signalling in rice
Source: Plant Biotechnol J. 2021 Aug 24;19(11):2304–18. doi: 10.1111/pbi.13661 (PMC8541776; doi:10.1111/pbi.13661)
Supplement: Supplementary file 13 — Figure S13. Relative expression of KNOX class 1 genes involved in panicle development in wild type and sd1 panicle branch primordia. Mean ± SE, n = 3. Differences to wild type plants indicated: *P < 0.05, **P < 0.01, t‐test. [file PBI-19-2304-s007.pptx]

## Slide 1
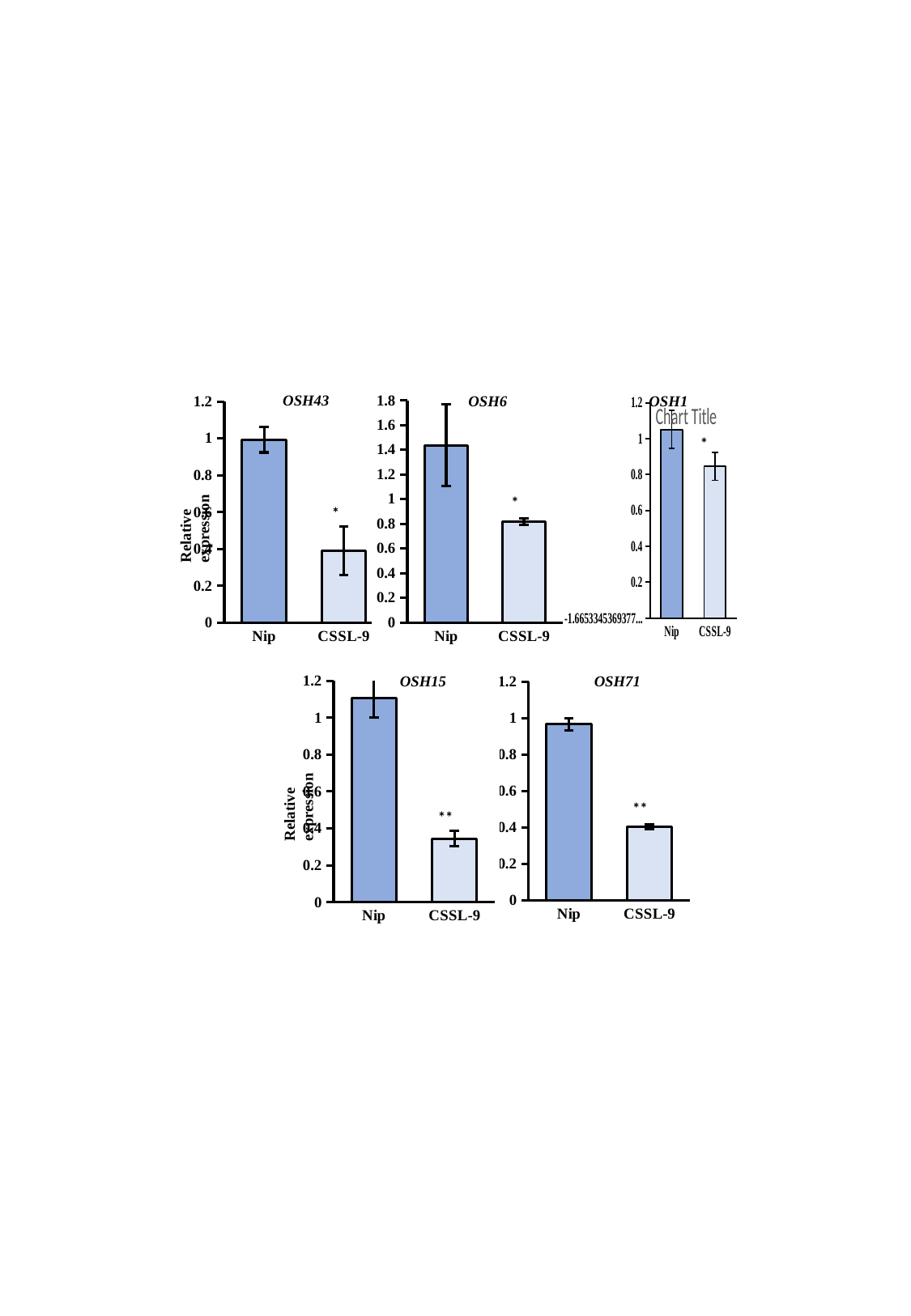

OSH43
OSH6
OSH1
### Chart
| Category | |
|---|---|
| Nip | 1.4359158908775773 |
| CSSL-9 | 0.8182582156490664 |
### Chart
| Category | |
|---|---|
| Nip | 0.9931163522466797 |
| CSSL-9 | 0.3913121196255378 |
### Chart:
| Category | |
|---|---|
| Nip | 1.0503551860600762 |
| CSSL-9 | 0.8448438883885935 |*
Relative expression
*
*
OSH71
OSH15
### Chart
| Category | |
|---|---|
| Nip | 1.1070974421975228 |
| CSSL-9 | 0.34473173020222164 |
### Chart
| Category | |
|---|---|
| Nip | 0.9665164957684033 |
| CSSL-9 | 0.4045127803788151 |Relative expression
**
**
